# Integrated miRNAs, Transcriptome, and Metabolome Uncover Underlying Mechanisms for Breast Muscle Metabolic Regulation in Liancheng White and Cherry Valley Ducks

**DOI:** 10.3390/ani16060934

**Published:** 2026-03-16

**Authors:** Linli Zhang, Xiaopan Liu, Li Li, Liang Huang, Zhiming Zhu, Zhongwei Miao, Nenzhu Zheng, Qingwu Xin

**Affiliations:** 1 Institute of Animal Husbandry and Veterinary Medicine, Fujian Academy of Agricultural Sciences, Fuzhou 350013, China; 2 Fujian Key Laboratory of Animal Genetics and Breeding, Fuzhou 350013, China

**Keywords:** duck, breast muscle, metabonomics, miRNA, amino acids, carbohydrates

## Abstract

Meat quality characteristics are important economic traits of ducks. To identify the molecular bases of these traits, we performed an integrated multi-omics analysis (metabolomics, transcriptomics, and miRNAomics) that compared the breast muscle of 300-day-old Liancheng white duck (LD), which is a lean-type breed prized for its soup flavor, and traditional meat duck Cherry Valley duck (CD), which is a fast-growing fat-type breed used for roasting. The results show that LD had higher levels of amino and bile acids, while CD had higher levels of carbohydrates. Integration analysis revealed key breed-specific molecular signatures. In LD, upregulation of the amino acid transporters *SLC7A6* and *SLC6A9* related to amino acid transport was consistent with elevated intramuscular amino acids. For carbohydrate metabolism, *SOCS3*—a well-established negative regulator of glucose uptake in mammalian skeletal muscle—was significantly upregulated in LD, consistent with their lower intramuscular carbohydrate levels. *SLC6A9* and *SOCS3* were predicted to be negatively regulated by oan-miR-1386. In LD, upregulation of the bile acid biosynthesis gene *CH25H* paralleled the higher bile acid content, suggesting complex, tissue-specific regulation of these pathways. This integrated analysis provides a resource for candidate genes, miRNAs, and metabolic pathways underlying breed-specific meat quality traits in ducks. The findings generate testable hypotheses for future functional studies and offer potential molecular targets for breeding strategies aimed at improving poultry meat quality.

## Simple Summary

This study aimed to identify specific metabolites, genes, and regulatory miRNAs associated with differences in meat quality and nutritional value between two duck breeds: the Liancheng white duck (LD) and a common meat-type Cherry Valley duck (CD). Using metabolomics, RNA-seq, and miRNA-seq, we compared the composition differences between their breast muscles. The results show that LD contained higher levels of amino and bile acids, while CD was richer in carbohydrates. Transcriptome analysis revealed differentially expressed genes (FDR < 0.05, |log_2_(Fold Change)| > 1) linked to these metabolic differences, including amino acid transporters (*SLC6A9*, *SLC7A6*), a carbohydrate metabolism regulator (*SOCS3*), and a bile acid synthesis gene (*CH25H*). Key regulatory miRNAs, such as oan-miR-1386 (which is predicted to target *SLC6A9* and *SOCS3*), were also identified. These findings provide a molecular foundation for improved meat quality and nutritional value from breeding ducks and offer candidate targets for future functional validation and marker-assisted selection.

## 1. Introduction

Different duck types show different meat quality traits. For example, a fat-type duck (e.g., Cherry Valley duck) is expected to prioritize metabolic pathways for energy storage and lipid deposition [1]. A lean-type duck (e.g., Muscovy duck) has high protein, polyunsaturated fatty acid, and low intramuscular fat contents [2]. Liancheng White Duck (LD) is characterized by “white feather, black mouth and feet”, with a mellow soup taste and no fishy smell or greasiness, especially in older ducks [3]; therefore, LD is often used for meat quality improvement in meat duck crossbreeding [4,5]. Research has shown that differences in lipids, proteins, chemicals, or metabolites can affect the flavor and structure of poultry meat [6]. Comparing the relationship between lipid metabolites and volatile flavor compounds in the breast muscle of Beijing You and Wenchang chickens showed that lysophosphatidylethanolamine (LPE) and lysophosphatidylcholine (LPC) synergistically promoted the formation of aldehyde flavor compounds with unsaturated fatty acids [7]. Volatiles (such as 2-nonanone, 2-nonanone, 3-hydroxymethyl, 2-methylheptanoic acid, caproic acid, and butyl ester) were formed through lipid oxidation and amino acid degradation pathways, shaping the difference in fat and grassy flavors between the two high-quality broilers (S3 and H lines) [8]. Although previous studies have documented differences in the volatile compounds in 65-day-old LD and 37-day-old CD meat and the differential metabolites between 120-day-old LD and CD meat [9,10], there is a lack of systematic understanding of the differences in metabolites and potential regulatory mechanisms in old ducks. Specifically, the integrated networks involving genes, miRNAs, and metabolites that coordinately determine breed-specific traits such as amino acid and fatty acid profiles are not well characterized.

miRNAs play important roles in regulating animal metabolism. They interact with mRNA, forming a complex molecular regulatory network [11]. By searching for miRNA target genes, we can understand their mechanisms and pathways, and thus, explore differentially expressed genes and key regulatory factors in the cells or tissues under different states. Non-coding RNAs can influence meat flavor by regulating the metabolic pathways and genes of flavor-related substances. For instance, miR-155-5p, identified in poultry studies, regulates fat synthesis by targeting PPARγ, thereby affecting the accumulation of flavor-related fatty acids [12]; similarly, miR-499-5p modulates glycolysis and fatty acid oxidation in broiler muscle [13]. Through multi-omics analysis, Wei Zhao identified three key mesoscopic networks, namely, gga–miR-1603–LNC_000324–PGM1, gga–miR-1768–LNC_000324–PGM1, and gga–miR-21–LNC_011339–AMPD1, which are closely related to inosine monophosphate (IMP) deposition, which is one of the important indicators of meat flavor [14]. Although these findings highlight the potential of miRNAs as potent regulators of muscle metabolism and composition in poultry, their specific roles and target networks in ducks—which exhibit distinct growth patterns, fat deposition, and meat quality traits—require direct investigation. In ducks, integrative omics approaches have begun to map miRNA–mRNA networks during embryonic and skeletal muscle development [15,16]. However, a critical gap exists in understanding how these regulatory molecules influence post-mortem meat quality attributes.

“Co-expression” analysis of differentially accumulated metabolite information and temporally expressed RNA can more effectively explore core regulatory networks and key candidate genes and clarify biological mechanisms. Therefore, this study selected the breast muscles of 300-day-old LDs and CDs, and then used metabolomic, miRNA-seq, and transcriptomic methods to explore the related genes’ effects on the meat quality and nutrients and their regulatory genetic mechanisms, providing a theoretical basis for improving duck meat quality.

## 2. Materials and Methods

### 2.1. Animals and Experimental Treatments

In this study, 1-day-old female Liancheng white ducklings and Cherry Valley ducklings were selected to feed and drink freely in a naturally lighted free-range enclosure. Diets with different nutritional levels were provided according to the nutritional needs of different growth stages; the diets’ nutritional components at each stage are shown in Table S1. After overnight fasting for 12 h, 10 ducks were selected from each group and slaughtered at 300 days old by captive-blot stunning and exsanguination. The breast muscle samples were taken from each breed for metabolite extraction, and three duck muscles were taken for mRNA and small-RNA sequencing. The samples were immediately frozen in liquid nitrogen and stored at −80 °C. The animal care and tissue collection were processed in accordance with the guidelines and regulations of the Animal Care and Use Ethics Committee of the Institute of Animal Husbandry and Veterinary Medicine, Fujian Academy of Agricultural Sciences (approval number: 20190901).

### 2.2. Metabolite Profiling Analysis

To better understand the differences in metabolites between the LD and CD breast muscles, samples from 10 ducks per breed (*n* = 10 biological replicates per group) were individually pretreated and determined using LC-MS/MS according to previous reports1 [10]. The samples were mixed in equal amounts to prepare QC samples. Three quality control (QC) samples were used to determine the instrument status and equilibrium chromatography–spectroscopy system and to evaluate the system’s stability during the whole experiment. By comparing the QC sample results from the total ion current (TIC), total sample principal component analysis (PCA), Hotelling’s T2 analysis (which can detect whether there are outliers), Pearson correlation analysis, and MCC test, the reproducibility and reliability of the data were guaranteed. An Ultra-High-Performance Liquid Chromatography (UHPLC) system from Agilent (Agilent Technologies, Santa Clara, CA, USA), coupled with a Waters HILIC chromatographic column (ACQUITY UPLC BEH Amide 1.7 mm, 2.1 × 100 mm column, Milford, MA, USA), was used to separate metabolites. The mobile phase comprised phase A (water containing 25 mM ammonium acetate and 25 mM aqueous ammonia) and phase B (acetonitrile) at a flow rate of 0.3 mL/min. The gradient elution program was as follows: 95% B, 0–1 min; 65% B, 1–14 min; 40% B, 14–16 min, 40% B from 18 to 18.1 min, 95% B from 18.1 to 23 min, and holding at 95% B. A Triple-TOF 6600 mass spectrometer (AB SCIEX, Framingham, MA, USA) was employed for the mass spectrometry analysis at Shanghai Applied Protein Technology Co. Ltd. (Shanghai, China).

The raw data were converted to the “mzXML” format using ProteoWizard MSConver software (ver 3.0.1). The ion peak alignment, retention time correction, and peak area extraction of metabolites were extracted using XCMS software (ver 3.8.2). Metabolite structure identification was performed using the in-house database (Shanghai Applied Protein Technology Co. Ltd., Shanghai, China). The structure of metabolites in biological samples was identified by matching with the retention time, molecular weight (molecular weight error within <25 ppm), secondary fragmentation spectrum, collision energy, and other information of metabolites in the local database, and the identification results were strictly checked and confirmed via a manual secondary check. The identification grade was above Level 2. After being preprocessed using Pareto-scaling, multivariate data analyses, including PCA and OPLS-DA, were conducted. The Variable Importance in Projection (VIP) value was calculated from the OPLS-DA model. For metabolites detected in both ESI+ and ESI− modes, the measurement from the mode with the higher content was reserved for the subsequent differential analysis. The volcano map of the metabolites was drawn using R software (ver 4.4.2). The significantly differentially accumulated metabolites (DAMs) were defined at fold change > 1.2, VIP > 1, and *p* value < 0.05. The selection of DAMs in this study was primarily driven by the combination of effect size (fold change) and multivariate model contribution (VIP), with the raw *p* value serving as a secondary filter. Therefore, no adjustment for multiple comparisons (e.g., FDR) was applied to the *p* values. Hierarchical cluster analysis was applied to significantly different metabolites to uncover variations in the metabolite concentrations between the groups. The clusterProfiler R package was employed to carry out a KEGG pathway enrichment analysis, and pathways were considered significant when the *p* value was less than 0.05.

### 2.3. Transcriptome Sequencing and Assembly Analysis

The total RNA was isolated using Trizol Reagent (Invitrogen, Carlsbad, CA, USA), while the RNA integrity was assessed using the Bioanalyzer 2100 system (Agilent Technologies, Santa Clara, CA, USA). Messenger RNA was purified with poly-T-oligo-attached magnetic beads (Invitrogen, Carlsbad, CA, USA). After fragmentation, the first strand cDNA was synthesized using random hexamer primers followed by second-strand cDNA synthesis. The cDNA libraries were sequenced on an Illumina Hiseq 2100 platform (LC Sceiences, Houston, TX, USA).

Before conducting genome assembly, low-quality sequencing reads were filtered out to obtain high-quality paired-end clean reads. The valid data were mapped to the duck reference genome (https://www.ncbi.nlm.nih.gov/genome/2793?genome_assembly_id=426073 (accessed on 23 October 2024) using Hisat 2.0. New transcripts were assembled and predicted using StringTie software (ver 2.2.3). Then, the new transcripts were annotated in Pfam, SUPERFAMILY, GO, KEGG, and other databases. The number of reads was normalized according to CPM (counts per million) using the edgeR package (ver 3.42.4) of the R language to correct the difference in sequencing depth between samples, and the coefficient of variation was evaluated. False discovery rate (FDR) control was implemented for all transcriptomic analyses using the Benjamini–Hochberg (BH) correction method via the topTags() function in the R package edgeR. Raw *p* values generated from differential expression analysis were adjusted with the false discovery rate (FDR) set at <0.05 to minimize the probability of type I errors and ensure the reliability of the identified differentially expressed transcripts. Significantly differential expression genes (DEGs) were selected based on the criteria of |log_2_(Fold Change)| > 1 and FDR < 0.05. This combination of thresholds is commonly used in the poultry transcriptome literature [17]. For visualizing, the R package was used to draw the DEGs heat map, scatter plot, and volcano map. Utilizing the clusterProfiler R package, enrichment analysis, including Gene Ontology (GO) and Kyoto Encyclopedia of Genes and Genomes (KEGG) pathways, was performed. Furthermore, significance enrichment was determined using a *p* value threshold of <0.05.

### 2.4. MicroRNA Sequencing and Identification

In the present experiment, six small-RNA libraries (three biological replicates for each breed) were constructed and processed on the Illumina SE50 platform (San Diego, CA, USA) in Novogene Co. Ltd. (Beijing, China). Raw sequencing data (raw reads) in fastq format were initially processed using Perl (ver 5.38.2) and Python (3.12.5) scripts. Clean reads were generated by filtering out low-quality reads, along with reads harboring poly-N stretches; reads contaminated with poly-N, 5′ adapter, or poly A or T or G or C; and reads without the 3′ adapter or the insert tag. The small-RNA tags were aligned to the reference sequence using Bowtie. The mapped small-RNA tags were further used to identify known miRNAs, with miRBase22.0 serving as the reference database. To exclude tags derived from protein-coding genes, repetitive sequences, rRNA, tRNA, snRNA, and snoRNA, the small-RNA tags were mapped against the RepeatMasker and Rfam databases and the reference genome.

### 2.5. Prediction and Functional Analysis of DE miRNAs Target Genes

To identify the differentially expressed known and novel miRNAs, the mapped small-RNA tags were used to look for known miRNAs. miRBase22.0 was used as the reference, and modified mirdeep2 (ver 2_0_0_5) software were used to obtain the potential miRNAs and draw the secondary structures. The available miREvo (ver 1.1) and mirdeep2 software were integrated to predict novel miRNAs. The differentially expressed miRNAs (DE miRNAs) were evaluated using the edgeR package. Significance was defined as |log_2_(Fold Change)| > 1 and *p* value < 0.05. The miRNA target gene was predicted using miRanda (ver 3.3a) and RNAhybrid (ver 2.0). A Gene Ontology (GO) enrichment analysis of the target genes was performed using the clusterProfiler R package (ver 3.8.1).

### 2.6. Construction and Functional Analysis of Potential DE miRNA–DEG Regulatory Network

The Pearson correlation coefficient was adopted to quantify the expression correlation between DE miRNAs and DEGs. Because miRNAs usually inhibit their target genes through incomplete complementary pairing, resulting in negative regulatory relationships, and |cor| < 0.3 usually means a weak correlation or no correlation, only the miRNA–mRNA interaction pairs with cor < −0.3 were selected for subsequent analyses. This threshold refers to previous studies on the interaction between non-coding RNA and target genes [18]. Under this threshold, the negative correlation is considered to have potential biological significance. The miRNA–mRNA network was visualized with Cytoscape software (ver 3.10.3). To investigate the potential function of the DE miRNA–DEG network in mediating duck breast muscle formation, DEGs from the miRNA–mRNA network were subjected to GO enrichment analysis, where results with *p* < 0.05 were regarded as statistically significant.

### 2.7. Co-Expression Analysis

For the integrated transcriptome, miRNA-seq, and metabolome analyses, DAMs and DEGs mapped into the same KEGG pathway were screened. Analysis of the co-expression network between miRNA, mRNA, and metabolites was performed with Cytoscape (version 3.7.2). Furthermore, a Pearson correlation analysis was performed on the DAMs and DEGs in the regulatory network.

### 2.8. Gene Expression and Validation Using qRT-PCR

To confirm the expression pattern results of high-throughput sequencing, six DEGs with high fold changes or that were metabolism-related and six miRNAs in the miRNA–mRNA regulatory network were analyzed using qRT-PCR. The total RNA was isolated from duck muscles with Trizol Reagent (Thermo Fisher, Waltham, MA, USA) and a PureLink miRNA Isolation Kit (Thermo Fisher, Waltham, MA, USA), following the manufacturer’s procedure. The specific primers of selected DEGs and miRNAs were designed via the Primer Premier 5.0 software, with all primer sequences presented in Table S2. GAPDH and U6 were used as the reference genes. These reference genes are widely validated as stably expressed in avian muscle tissues and have been commonly used as internal controls in duck molecular biology studies [19]. The primers were synthesized by Bioengineering (Shanghai) Co., Ltd. (Shanghai, China). qRT-PCR reaction system (20 μL): Power SYBR Green Master Mix 10 μL (Applied Biosystems, Foster City, CA, USA), SDW 8.0 μL, 1 μg/μL cDNA 1.0 μL, 10 μmol/L upstream primers 0.5 μL, and 10 μmol/L downstream primers 0.5 μL; reaction conditions: 95 °C for 1 min, 95 °C for 15 s, and 60 °C for 25 s, with 40 cycles. The relative expressions of genes were calculated using the 2^−ΔΔCt^ method.

## 3. Results

### 3.1. Difference Between Metabolites in Duck Breast Muscle

In total, 8691 and 8593 ion peaks were obtained in ESI+ and ESI− modes, respectively. To ensure the reliability of non-targeted metabolomics data, the total ion chromatograms of quality control samples were overlapped and compared. The results show that the response intensity and retention time of each chromatographic peak basically overlapped (Figure S1A), indicating that the instrument had good stability. The PCA diagram shows that the QC samples were tightly aggregated in the positive and negative ion modes, indicating the good repeatability of the LC-MS analysis during the experiment (Figure S1B). Hotelling’s T2 analysis showed that all samples had no outliers within the 99% confidence interval (Figure S1C). Pearson correlation analysis was performed on the QC samples, and the correlation coefficient was greater than 0.9, indicating a good correlation (Figure S2A). The MCC test showed that the QC sample had less volatility in the range of positive and negative standard deviations, and the data could be used for subsequent analysis (Figure S2B).

**Figure 1:**
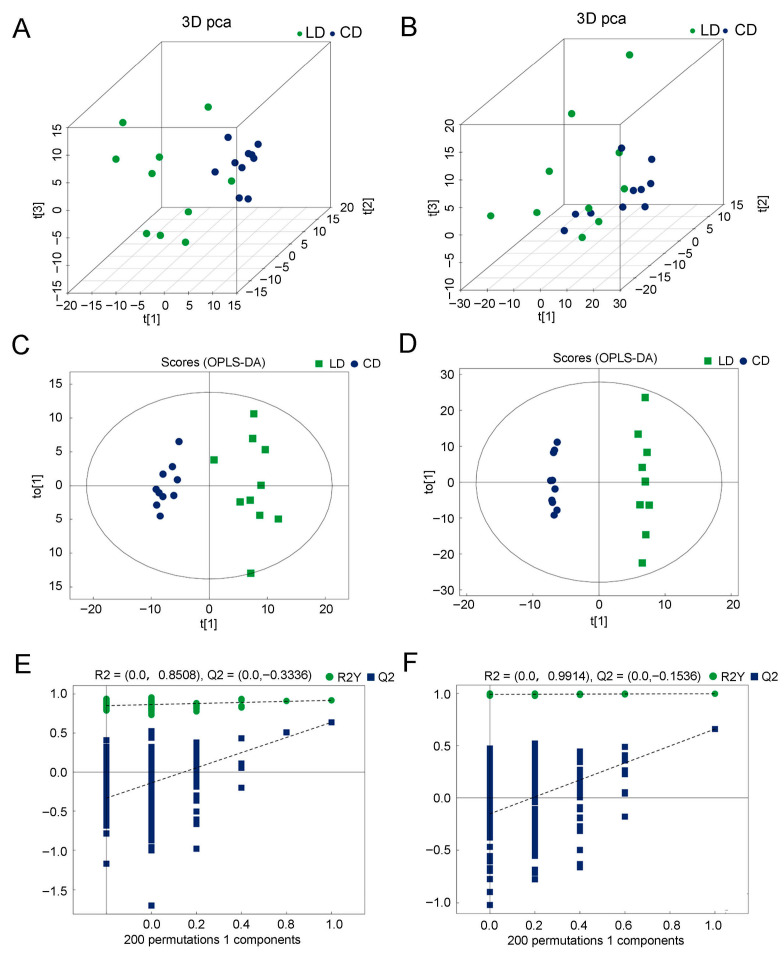
The principal component analysis score (**A**,**B**), OPLS-DA score (**C**,**D**), and cross-validation (**E**,**F**) plots generated from the OPLS-DA models. OPLS-DA score plots (R^2^ X = 0.3, R^2^ Y = 0.919, Q^2^ = 0.637, ESI+; R^2^ X = 0.63, R^2^ Y = 0.997, Q^2^ = 0.659, ESI−); cross-validation plot of OPLS-DA model with 200 permutation tests (R^2^ = 0.8508, Q^2^ = −0.336, ESI+; R^2^ = 0.9914, Q^2^ = −0.1536, ESI−).

Following a data integrity check and normalization, an unsupervised PCA (Figure 1A,B) was conducted before supervised OPLS-DA (Figure 1C,D). In the OPLS-DA score plot, the breast muscle compositions of LD and CD at 300 days old (Figure 1C) were clustered together, indicating that the established mathematical model was reliable. The 200 permutation tests show that all Q points from left to right were lower than the original blue Q2 points at the rightmost end, indicating that the model was robust and reliable, and there was no overfitting (Figure 1E,F). The OPLS-DA and two-hundred-permutation tests demonstrated the robustness and reliability of the constructed model, which excluded the possibility of overfitting.

**Figure 2:**
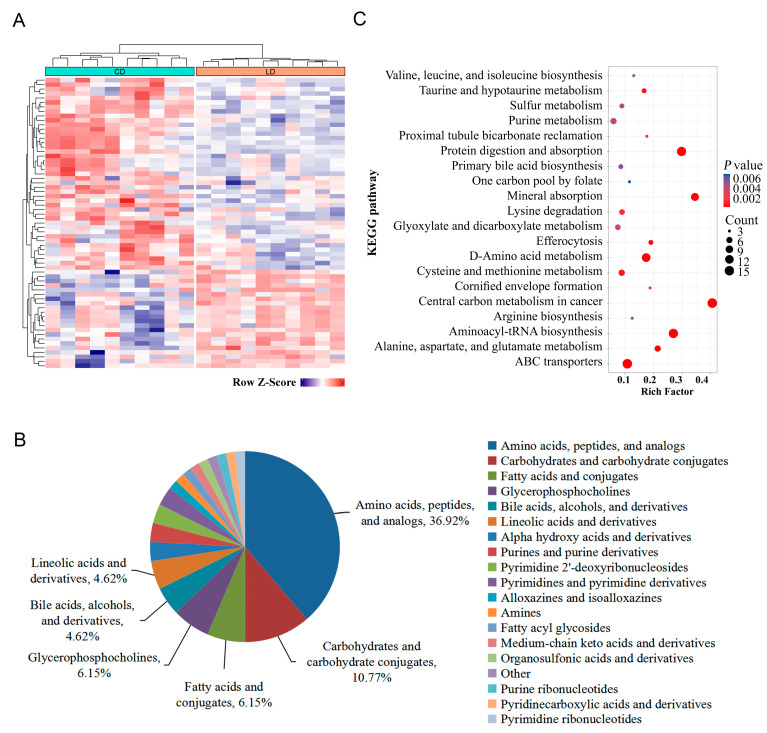
Significantly differential abundant metabolites (DAMs) in LD and CD muscles. (**A**) Heatmap of DAMs. The color indicates the level of relative content of each DAM, from dark purple (high) to red (low). (**B**) Classification of the significant DAMs. The percentage represents (the number of significant DAMs in a sub class/the total number of significant DAMs) × 100%. (**C**) KEGG enrichment analysis of significant DAMs.

A total of 27 and 38 statistically significant DAMs were identified in ESI+ and ESI- modes between the two breeds. The hierarchical cluster analysis showed that about three-quarters of the DAMs were accumulated in the breast muscle of LD (Figure 2A). In the LD group, 42 out of 65 significant biomarkers were found to be upregulated while 23 were downregulated. A total of 18 subclasses were assigned to these significant DAMs. The percentage contribution of each subclass (Figure 2B) was calculated based on the count of significant DAMs belonging to that subclass relative to the total number of significant DAMs (n = 65). Among them, the major subclasses included amino acids, peptides, and analogs (36.92%); carbohydrates and carbohydrate conjugates (10.77%); glycerophosphocholines (6.15%); fatty acids and conjugates (6.15%); and bile acids, alcohols, and derivatives (4.42%).

The largest fold differences in the upregulated metabolites in the LDs were found for taurocholate, inosine, and taurochenodeoxycholate, while in the CDs, they were found for D-mannose-6-phosphate, adenosine 5′-phosphate sulfate (APS), and D-glucose 6-phosphate. Out of 24 amino acids, peptides, and analogs, 21 of them were higher in Liancheng duck white, such as seven essential amino acids (L-methionine, L-threonine, L-lysine, L-phenylalanine, L-histidine, L-valine, and L-leucine) and umami amino acids (L-glutamate, glycine, and L-alanine) (Table 1). Meanwhile, all seven carbohydrates and carbohydrate conjugates were upregulated in CD, including D-glucose 6-phosphate, D-mannose-6-phosphate, D-erythrose 4-phosphate, D-glucosamine 6-phosphate, D-mannose 1-phosphate, alpha-D-glucose, and D-ribulose 5-phosphate.

In addition, bile acids, alcohols, taurine derivatives (taurocholate, taurochenodeoxycholate, taurodeoxycholic acid), and taurine had higher levels in the LD muscle, with fold changes of 16.69, 6.00, 5.12, and 1.98 (LC vs. CD), respectively. Among the fatty acids and conjugates, the content of stearic acid was higher in LD, while the levels of adipic acid were higher in CD. All four glycerophosphocholines were higher in LD. Overall, the amino and bile acids were maintained at relatively high levels in the muscles of 300-day-old LDs, and carbohydrates and carbohydrate conjugates were high in the CD muscle.

The KEGG pathway analysis showed (Figure 2C) that these differential metabolites were enriched in 92 signaling pathways. Among them, 17 pathways were significantly enriched, including protein digestion and absorption; ABC transporters; aminoacyl-tRNA biosynthesis; and alanine, aspartate, and glutamate metabolisms. DAMs were also enriched in multiple pathways related to amino acid, carbohydrate, and bile acid metabolisms, such as D-amino acid metabolism; alanine, aspartate, and glutamate metabolisms; arginine biosynthesis; glycolysis and gluconeogenesis; fructose and mannose metabolisms; primary bile acid biosynthesis; and taurine and hypotaurine metabolisms. These pathways may play important roles in regulating the differences between metabolites in duck breast muscle, which, in turn, affect the formation of breast muscle quality.

### 3.2. Screening of Differentially Expressed mRNAs

**Figure 3:**
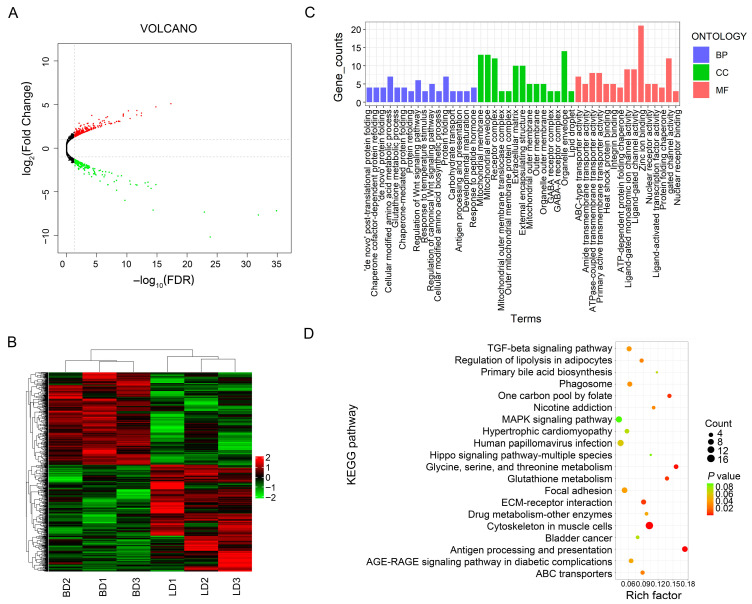
Overview of differentially expressed genes (DEGs) and functional annotation in duck chest muscles. (**A**) Volcano plot of differentially expressed mRNAs. Dots in different colors represent significantly upregulated (red), downregulated (green), and non-significant (black) miRNAs. The dashed lines indicate the significance thresholds. (**B**) Heat map of differentially expressed mRNAs. (**C**) Gene Ontology (GO) terms enrichment analysis of DEGs. (**D**) KEGG enrichment analysis of DEGs.

Following the filtration of low-quality reads and removal of adaptor sequences, an average of 45,256,070 high-quality clean reads were yielded across the biological replicates. The GC contents of the clean sequencing data varied from 51.59% to 53.62%, and the Q20 and Q30 quality scores reached over 98.69% and 96.31%, respectively. All clean reads were aligned to the duck genome, and the mapping rates were between 79.22% and 81.93% (Table S3). Transcriptome assembly and alignment further identified 19,520 mRNAs in the duck muscle, including 19,310 known transcripts and 210 novel mRNAs. Based on the criteria of FDR < 0.05 and|log_2_(Fold Change)| ≥ 1, 465 DEGs were screened out in the LD muscle compared with the CD muscle (Table S4). The expression patterns of DEGs were visualized via volcano plots and heat maps, which revealed 266 upregulated DEGs and 199 downregulated DEGs in the LD group (Figure 3A,B).

### 3.3. Functional Enrichment of the Differentially Expressed mRNAs

The Gene Ontology (GO) analysis showed that 261 DEGs were functionally annotated (Figure 3C). Among those DEGs, in the “molecular function” category, 21 and 19 genes were in “zinc ion binding” and “ATP-dependent activity”, respectively. In the “cellular component” category, the major genes were in “emitochondrial envelopee” (13) and “mitochondrial envelope” (13). In the “biological process” category, 14 and 13 were concentrated in “cell development” and “protein-containing complex assembly”, respectively. Seven genes were enriched in the top category of “ABC-type peptide transporter activity”. Several enriched items were related to the metabolic pathways underlying the breed-specific metabolite profiles observed in this study. These included the L-amino acid biosynthetic process, with genes such as *ASNS* (asparagine synthetase, catalyzing aspartate → asparagine) and *SHMT1* (serine hydroxymethyltransferase, catalyzing serine ↔ glycine interconversion); amino acid transmembrane transport, with transporters including *SLC6A9* (encoding glycine transporter GlyT-1) and *SLC7A6* (encoding the cationic/neutral amino acid exchanger y+LAT2); lipid transporter activity, including *ABCA7*, *MFSD2A*, and *SLC27A3*; and carbohydrate metabolic process, including *HK2* (hexokinase 2), *TALDO1* (transaldolase), *GLB1L*, and *IDUA*.

The KEGG pathway analysis revealed that DEGs were assigned to 242 pathways (Figure 3D), among which 14 pathways were significantly enriched, such as the cytoskeleton in muscle cells; glycine, serine, and threonine metabolisms; and ABC transporters. Non-significant enriched pathways related to carbohydrate and bile acid metabolisms were also screened, such as “glycolysis/gluconeogenesis” (involving *HK2*/*LOC101801299*), “insulin signaling pathway” (*FOXO1*/*SH2B2*/*SOCS3*/*HK2*), and primary bile acid biosynthesis (*CH25H*/*AKR1D1*). These pathways and genes may affect the accumulation of amino acids, carbohydrates, and bile acids in duck breast muscle. Further joint analysis with the metabolome was conducted and the results are provided below.

Critically, several of these DEGs encoded proteins with direct catalytic or transport functions that provide mechanistic links to the observed metabolite differences. The upregulation of amino acid transporters *SLC6A9* and *SLC7A6* in LD offered a direct mechanism for the elevated intramuscular levels of glycine and other amino acids detected in the metabolome. Additionally, differential expression of the metabolic enzyme *SHMT1* may fine-tune the balance between serine and glycine. The downregulation of *HK2*—the rate-limiting enzyme of glycolysis catalyzing glucose → glucose-6-phosphate—is consistent with the significantly lower levels of glycolytic intermediates (e.g., glucose-6-phosphate, mannose-6-phosphate) observed in the LD muscle. The upregulation of *CH25H*, which initiates an alternative pathway of bile acid synthesis, aligns with the elevated levels of taurine-conjugated bile acids in LD. Meanwhile, other DEGs, such as the insulin-signaling regulator *SOCS3*, may influence glucose uptake.

### 3.4. Identification of the Differentially Expressed miRNAs

**Figure 4:**
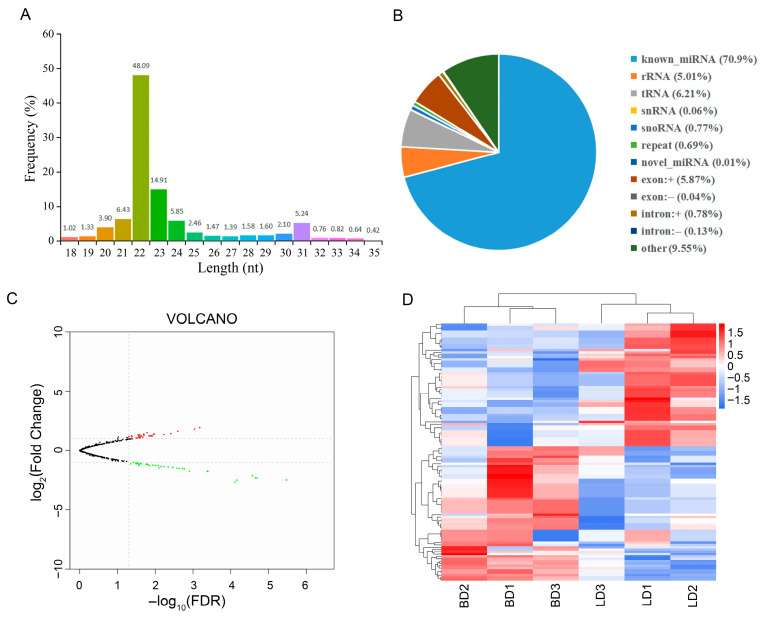
Overview of small-RNA sequencing in the duck muscles. (**A**) Size distribution of all miRNAs. The X-axis depicts their length (nt), and the Y-axis represents the frequency (%). (**B**) Proportions of small-RNA types in the clean reads. (**C**) Volcano plots of differentially expressed miRNAs. Dots in different colors represent significantly upregulated (red), downregulated (green), and non-significant (black) miRNAs. The dashed lines indicate the significance thresholds. (**D**) Heat map of differentially expressed miRNAs.

A total of 70,784,119 clean reads were generated from the six constructed libraries, corresponding to a high ratio over 97.9% (Table S5). The clean reads with lengths ranging from 18 to 35 nt were used for the subsequent analysis. The frequency of sRNA fragments with different lengths is shown in Figure 4, where the longest miRNA was 22 nt (Figure 4A). After the read mapping and novel miRNA prediction, 70.9% of clean reads were identified as known miRNAs; 0.01% were novel miRNAs; and the remaining reads (29%) included rRNAs, tRNAs, snRNAs, and snoRNAs (Figure 4B). In total, 1475 miRNAs were identified across the six libraries, which included 1431 known miRNAs and 44 novel miRNAs. To identify the differentially expressed miRNAs, we initially applied a cutoff of FDR < 0.05, which yielded very few significant candidates. Given the strictness of the multiple testing correction and the relatively subtle expression changes in this study, thresholds of *p* < 0.05 and |log_2_(Fold Change)| ≥ 1.0 were adopted for downstream bioinformatic analysis to retain biologically meaningful candidates; a total of 111 DE miRNAs met these thresholds. Compared with the CD group, 53 DE miRNAs were significantly upregulated and 58 were downregulated in the LD group (Figure 4C). A heat map was constructed to characterize the expression profiles of these DE miRNAs (Figure 4D).

### 3.5. Functional Annotation and the DE miRNA–DEG Regulatory Network Construction

**Figure 5:**
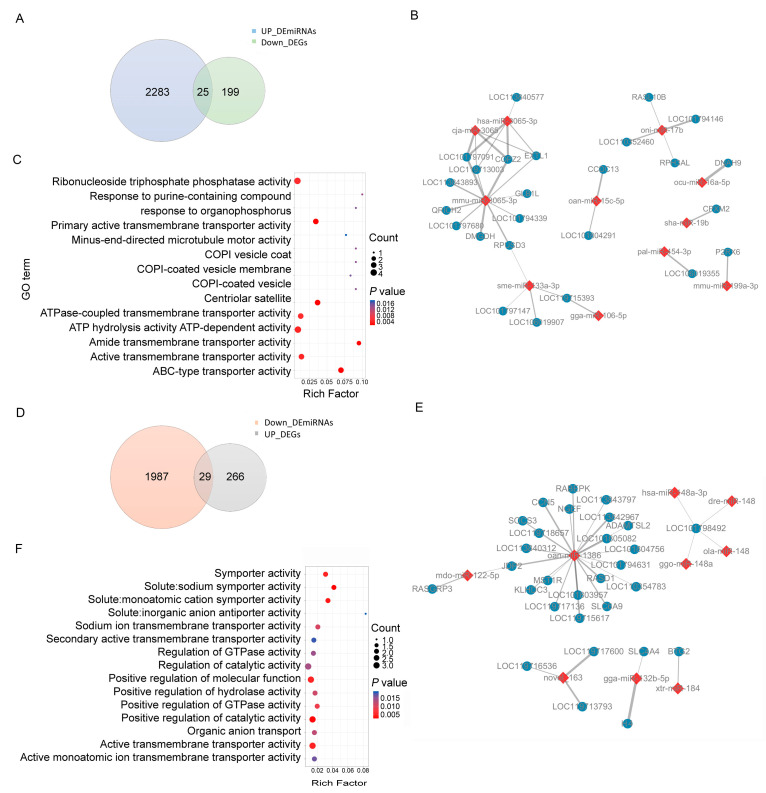
Identification of the DE miRNA–DEG network. (**A**) Venn diagram showing the common genes that simultaneously belong to the downregulated DEGs and the predicted target genes of upregulated DE miRNAs. (**B**) Up_DE miRNA–Down_DEG regulatory network. Red squares and blue circles indicate Up_DE miRNAs and Down_DEGs, respectively. (**C**) GO enrichment analysis for Up_DE miRNA–Down_DEG regulatory network. (**D**) Venn diagram showing the common genes that simultaneously belong to the upregulated DEGs and the predicted target genes of Down_DE miRNAs. (**E**) Down_DE miRNA–Up_DEG regulatory network. Red squares and blue circles indicate Down_DE miRNAs and Up_DEGs, respectively. (**F**) GO enrichment analysis for Down_DE miRNAs and Up_DEGs regulatory network.

To better understand the function of the DE miRNAs, we first predicted the target genes of the upregulated and downregulated DE miRNAs, respectively, using RNAhybrid and miRanda software. A total of 2254 overlapped genes were identified as the upregulated DE miRNAs target genes. Through expression correlation analysis (cor < −0.3), 11 existing DE miRNAs were found to target 25 genes, forming 35 miRNA–mRNA interaction pairs (Table S6, Figure 5A,B). Among these DE miRNAs, mmu-miR-3065-3p was recognized as a core hub miRNA. To further investigate the potential molecular mechanisms by which miRNAs regulated duck muscle metabolism, GO enrichment analyses were performed on the target DEGs of these DE miRNAs (Figure 5C). The shared DEGs in the regulatory network show significant (*p* < 0.05) enrichment in 42 biological processes (Table S7), such as ABC-type transporter activity (*LOC101797091*/*LOC101797680*/*LOC101794339*) and response to purine-containing compound (*P2RX6*).

Furthermore, 1948 genes were identified as target genes of downregulated DE miRNAs (Figure 5D). With cor < −0.3 as the consistent screening condition, a regulatory network was built, which comprised 33 miRNA–mRNA pairs (Table S6) consisting of 9 DE miRNAs and 29 DEGs (Figure 5E). Among these miRNAs, oan-miR-1386 presented the highest connectivity degree in the network. GO functional enrichment revealed significant enrichment (*p* < 0.05) of the upregulated DEGs in the regulatory network in 97 biological processes (Table S8), including solute:sodium symporter activity (*SLC6A9*), regulation of intracellular pH (*SLC4A4*), and phosphatidylinositol 3-kinase complex (*SOCS3*) (Figure 5F).

### 3.6. mRNA–miRNA Metabolites Co-Expression Network

**Figure 6:**
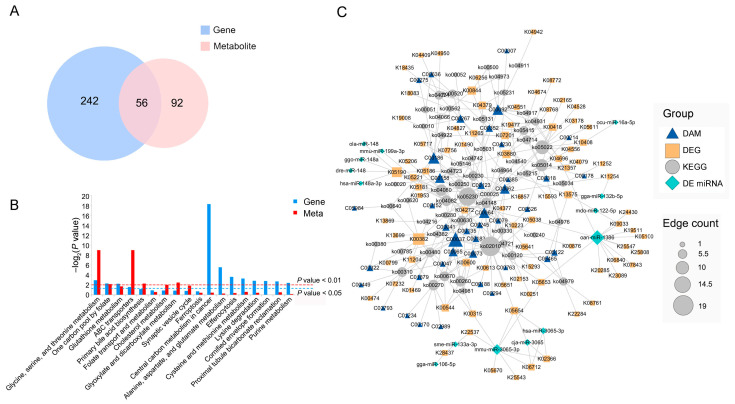
Integrated transcriptome, miRNA-seq, and metabolome analysis. (**A**) Venn diagram showing the common pathway between the transcriptome and metabolome. (**B**) Co-enriched pathway of DEGs and DAMs (partly). (**C**) DE miRNA–DEG–DAM network.

KEGG pathways, together with DEGs and DAMs from transcriptomic and metabolomic analyses, were subjected to integrated analysis. In total, 56 pathways were co-enriched and four pathways were significantly enriched (*p* < 0.05) (Figure 6A,B). The co-enriched DEGs and DAMs and the DEmiRNA were integrated using Cytoscape (Figure 6C). In the co-expression network, 11 miRNAs and 11 target genes were screened, involving 14 metabolic pathways (Table 2). Furthermore, correlation analysis was conducted on the 11 targeted DEGs and co-enriched DAMs (Table S9). Among the nodes, oan-miR-1386 was predicted to target the *SLC6A9* gene (which encodes a glycine transporter) and co-express with glycine and L-glutamate in neuroactive ligand signaling and synaptic vehicle cycle pathways. Correlation analysis revealed that the expression of SLC6A9 was significantly positively correlated with its substrate, i.e., glycine (Pearson’s r = 0.857, *p* < 0.05). This places oan-miR-1386 and *SLC6A9* as key nodes within the neuroactive ligand–receptor interaction pathway, allowing us to propose a hypothesis where miRNA-mediated post-transcriptional regulation of the transporter could influence amino acid availability. Concurrently, oan-miR-1386 was also predicted to target *SOCS3*, a key inhibitor of insulin signaling; notably, SOCS3 expression showed a strong negative correlation with alpha-D-glucose (Pearson’s r = −0.924, *p* < 0.01). This suggests a potential role for the oan-miR-1386/SOCS3 axis in modulating insulin resistance pathways that intersect with carbohydrate metabolism. In summary, our integrative analysis suggested that oan-miR-1386 serves as a pleiotropic regulator, potentially linking amino acid neurotransmitter metabolism (via SLC6A9) and insulin signaling (via SOCS3) to drive the distinct muscle metabolic profiles observed between the two duck breeds.

### 3.7. qRT–PCR Validation

**Figure 7:**
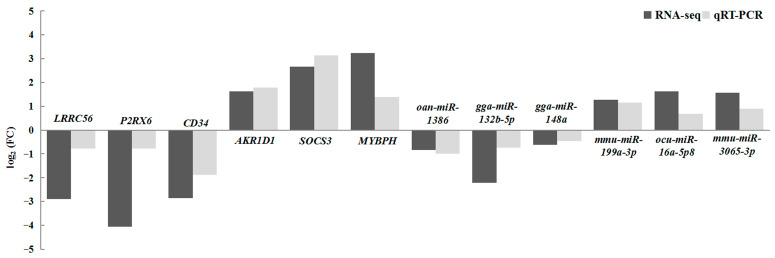
Verification of miRNA-seq and mRNA-seq results via qRT-PCR.

For verification of the RNA-seq data reliability, qRT-PCR assays were performed to determine the relative expression levels of six differentially expressed genes and six miRNAs in the miRNA–mRNA regulatory network in the LC and CD groups. The results show that the relative expressions of upregulated and downregulated genes were consistent with the high-throughput sequencing results (Figure 7), indicating that the transcriptome data was reliable.

## 4. Discussion

Liancheng white duck is a small local duck breed; after long-term closed breeding, it has excellent characteristics, such as unique flavor and rich nutrition. Meanwhile, CD is a large meat duck. In this study, joint multi-omics analysis was used to study the regulatory genes and miRNAs of meat flavor differences between LD and CD to provide a reference for the breeding of excellent local duck varieties and meat quality evaluation.

Metabolomics analysis showed that the amino and bile acids in the muscle of 300-day-old LD were maintained at a relatively high level, while the carbohydrate content in the muscle of CD was higher. The carbohydrate levels were similar to those at 120 days old, but the amino acids were different [10]. This may be related to age; some studies showed that age was closely related to the deposition of flavor substances and nutrients [20]. The lipid, protein, and metabolite contents in livestock and poultry muscles can affect the meat quality and flavor. The composition of these metabolites may partially explain its traditional culinary reputation for producing soup with a mellow taste and reduced greasiness, as higher amino and bile acid contents are generally associated with richer flavor and nutritional value [21]. However, as our study focused on metabolic profiling, direct correlations with sensory perception remain to be established. Future studies integrating metabolomics with controlled sensory panels or taste activity value (TAV) calculations are needed to confirm whether these metabolite differences translate to perceptible flavor differences.

The essential and flavor amino acids, such as L-glutamate, L-asparagine, glycine, and L-alanine, accumulated more in the breast muscle of LD, which may enhance the umami taste of LD [9], while threonine, serine, glycine, and alanine can increase the sweetness [22]. L-arginine and L-lysine were found to be more abundant in LD muscle tissue than in Mianyang Shelduck muscle [23]. The glutamate, aspartic acid, and arginine contents in breast muscle of slow-growth ducks are higher than those of fast-growth ducks [6].

Integrated analysis showed that amino-acid-metabolism-related genes, such as *SLC7A6* and *SLC6A9*, were significantly upregulated in the LD breast muscle. This suggests that *SLC7A6* and *SLC6A9* may play a synergistic role in regulating the homeostasis of amino acid metabolism in LDs. *SLC7A6* (corresponding protein y+LAT) was mainly involved in the bidirectional transmembrane transport of basic and neutral amino acids. It exports basic amino acids, such as L-arginine and lysine, from the inside of the cell and exchanges with neutral amino acids—such as L-leucine, L-glutamine, and isoleucine—and sodium ions outside the cell [24,25]. Knockdown of *SLC7A6* in mouse muscle cells significantly inhibits the outward transport of arginine in the presence of lysine, methionine, glutamine, or leucine [26]. The expression level of *Slc7a6* in liver tumor cells was significantly upregulated, accompanied by enhanced arginine uptake [27]. *SLC7A6* upregulation is likely to promote the absorption and accumulation of these amino acids in LD.

*SLC6A9* encodes glycine transporter-1 GlyT-1, which can transport extracellular glycine into cells. GlyT1 is mainly expressed in astrocytes and glutamatergic neurons, and is also widely distributed in the intestine, oocytes, early embryos, and muscles [28,29,30]. Consistent with this conserved function, we observed a significant positive correlation between *SLC6A9* expression levels and glycine content across individual duck samples (Pearson’s r = 0.837, *p* < 0.05), suggesting that elevated *SLC6A9* expression may contribute to glycine accumulation in LD muscle. In other systems, GlyT-1 was shown to promote satellite cell proliferation and muscle regeneration by actively transporting glycine into satellite cells [31]. Meanwhile, imported glycine can be converted to L-serine via serine hydroxymethyltransferase (*SHMT*) [32], which was also highly expressed in LD. While these mechanistic insights from mouse and human studies provide plausible hypotheses, the functions in ducks still need to be further verified.

*SLC6A9* expression can be regulated by miRNA. Esperanza Jiménez found that miR-96 negatively regulated the expression of *SLC6A9* in the retina [33]. bta-miR-140 is a target for *SLC6A9* in fertile bull semen [34]. In our co-expression network, oan-miR-1386 was predicted to target *SLC6A9*. Although a similar sequence has been found in human plasma and bovine fetuses [35,36], its function remains uncharacterized. Our data thus generated the testable hypothesis that oan-miR-1386 modulates *SLC6A9* expression and thereby influences glycine homeostasis in duck breast muscle. Future studies employing loss- and gain-of-function approaches in primary duck myocytes—combined with targeted metabolomics to measure glycine uptake flux—are required to establish causality and confirm whether the regulatory axis predicted here is functionally operative in ducks.

Metabolomics analysis revealed that CD muscle exhibited significantly higher levels of several carbohydrates, including D-glucose 6-phosphate, alpha-D-glucose, D-mannose-6-phosphate, D-mannose 1-phosphate, and D-glucosamine 6-phosphate. The accumulated G6P and its derivatives (especially glucosamine-6-phosphate) are the flavor precursors of the Maillard reaction [37,38]. During cooking, they can react with amino acids in meat to produce the unique strong aroma and brownish-red color of Beijing roast duck. This is similar to the reports on some fast-growing chickens. When comparing the S-line (fast-growing) and D-line (egg-type) of Guangxi partridge chickens, it was found that the α-D-glucose, α-D-galactose 1-phosphate, fructose, D-mannose 6-phosphate, D-mannose 1-phosphate, and D-fructose-6-phosphate levels in S-line chickens increased and were enriched in amino sugar and nucleotide sugar metabolic pathways [39]. Huo et al. compared fast- and slow-growing chicken breeds and found that fast-growing chickens had higher concentrations of glycogen, glucose, 6-phosphate glucose, and lactic acid and a higher glycolytic potential [40]. These differences reflect the different susceptibility of different duck breeds to grape metabolism, as well as the variety specificity in muscle glucose metabolism and energy utilization strategies [41]. CD is a typical “fast large meat duck”, which may need to increase muscle and energy rapidly, and thus, the intermediate products of glucose metabolism accumulate.

*SOCS3* was significantly upregulated while carbohydrates were downregulated in LD muscle, and a negative correlation between *SOCS3* and alpha-D-glucose (Pearson’s r = −0.924, and *p* < 0.01) was found. These findings align with the established role of *SOCS3* in mammalian systems [42,43]. *SOCS3* (cytokine signaling inhibitor 3) is a key molecule in the metabolic regulatory network. In mammals, it can aggravate insulin resistance and regulate glucose and lipid homeostasis by inhibiting the JAK/STAT signaling pathway, interfering with fat differentiation and energy metabolism [44]. In rodents, muscle-specific overexpression of *SOCS3* directly impairs IRS-1/Akt signaling and reduces insulin-stimulated glucose uptake [45]. Conversely, muscle-specific deletion of *SOCS3* enhances skeletal muscle glucose uptake and protects against diet-induced insulin resistance [42]. In humans, *SOCS3* mRNA expression in skeletal muscle is significantly negatively correlated with insulin-stimulated glucose uptake in patients with type 2 diabetes [46]. *SOCS3* expression was subject to multi-level regulation, encompassing transcription, post-transcriptional, post-translational, and epigenetic levels [47]. In mice, miR-152 affected hepatic insulin resistance in gestational diabetes by targeting *SOCS3* [48]. The upregulated expression of miR-155 in hepatic tissue improved lipid metabolism homeostasis in diabetic mice by suppressing the expression of target gene *SOCS3* [49]. In our co-expression network, *SOCS3* was predicted to be negatively regulated by oan-miR-1386. Therefore, our integrated multi-omics data generate the testable hypothesis that oan-miR-1386-mediated suppression of *SOCS3* contributes to enhanced insulin sensitivity and glucose utilization in LD muscle, resulting in lower carbohydrate accumulation. When employing oan-miR-1386 knockdown/overexpression in a relevant cell line (e.g., duck myocytes), including corresponding metabolite levels is essential to validate this proposed regulatory mechanism and establish causality.

The bile acid content, such as taurocholate, taurochenodeoxycholate, and taurine, in the LD muscle was higher. It has been reported that taurochenodeoxycholate is the main bile acid type in poultry [50]. The main site of bile acid biosynthesis is the liver, but other tissues, such as the lung, skeletal muscle, and adipose tissue, can also synthesize it in small amounts [51,52]. In addition to being involved in energy metabolism in muscles, adipose tissue, and the liver, taurine-conjugated bile acids also have antioxidant and anti-inflammatory effects [53,54]. Among the differentially expressed genes in the primary bile acid biosynthesis pathway, *CH25H* (cholesterol 25-hydroxylase) was significantly upregulated in LD breast muscle. Although its canonical role in hepatic bile acid synthesis is well-established [50], *CH25H* expression and function are not liver exclusive. In chicken muscle, *CH25H* gene exhibits a high expression during early muscle development [55]. *CH25H* has been identified in mouse skeletal muscle, where it is induced by TNF-α, and its product, i.e., 25-HC, directly regulates muscle cell metabolism and atrophy [56]. Whether *CH25H* plays a direct role in intramuscular bile acid metabolism in ducks remains unknown. The upregulation of *CH25H* in LD muscle may reflect local bile acid production with potential signaling or metabolic functions, or it may be an indirect correlate of systemic metabolic differences between breeds, although this hypothesis requires direct experimental testing in avian species.

## 5. Conclusions

In summary, breed-specific metabolite profiles in the breast muscle of 300-day-old Liancheng white duck and Cherry Valley duck were identified, with LD showing higher levels of amino and bile acids, and CD being enriched in carbohydrates. Multi-omics integrative analysis further revealed differentially expressed genes (e.g., *SLC7A6*, *SLC6A9*, *SOCS3*, *CH25H*) and miRNAs (e.g., oan-miR-1386) associated with these metabolic variations and thus were proposed as candidate regulatory factors. These findings provide novel insights into a system-level resource for understanding the molecular basis of duck meat quality and offer testable hypotheses and potential molecular targets for breeding strategies aimed at meat quality improvement in poultry.

## Figures and Tables

**Table 1:** Majority categories distinguishing metabolites in breast muscles between two breeds.

	Name	*m*/*z*	rt(s)	VIP	Fold Change (LD/CD)	*p* Value	Subclass
1	D-proline	116.07	633.21	4.92	4.78	3.73 × 10^−3^	Amino acids, peptides, and analogues
2	DL-methionine sulfoxide	166.05	738.22	1.39	3.42	4.60 × 10^−4^	
3	L-saccharopine	277.14	893.11	1.35	3.11	2.30 × 10^−3^	
4	L-methionine	150.06	572.44	3.60	2.62	1.28 × 10^−4^	
5	L-threonine	118.05	649.67	2.60	2.62	1.78 × 10^−5^	
6	N-acetyl-DL-methionine	190.05	352.45	1.02	2.47	1.43 × 10^−3^	
7	1-aminocyclopropanecarboxylic acid	84.04	747.49	2.33	2.14	1.98 × 10^−2^	
8	L-asparagine	131.05	690.23	2.18	1.98	6.05 × 10^−4^	
9	L-arginine	173.10	931.15	2.05	1.96	1.79 × 10^−2^	
10	L-lysine	145.10	956.63	2.13	1.93	1.55 × 10^−3^	
11	L-histidine	154.06	699.52	1.61	1.86	6.04 × 10^−3^	
12	DL-serine	104.03	689.47	2.58	1.86	1.82 × 10^−2^	
13	L-glutamate	148.06	805.23	2.71	1.80	6.07 × 10^−3^	
14	Lphenylalanine	164.07	459.36	3.24	1.68	4.81 × 10^−2^	
15	L-glutamine	145.06	685.20	8.74	1.67	1.87 × 10^−2^	
16	L-pyroglutamic acid	147.08	787.89	2.30	1.55	2.36 × 10^−2^	
17	L-leucine	130.09	494.04	3.26	1.50	2.55 × 10^−2^	
18	L-valine	116.07	548.47	1.80	1.40	6.32 × 10^−3^	
19	Glycine	74.02	667.32	1.11	1.39	3.99 × 10^−3^	
20	L-alanine	88.04	672.08	2.28	1.37	3.03 × 10^−2^	
21	His-Pro	235.12	282.45	1.06	1.24	3.53 × 10^−3^	
22	3-methylhistidine	170.09	869.47	1.05	0.66	5.94 × 10^−5^	
23	N6,N6,N6-Trimethyl-L-lysine	189.16	1010.27	1.42	0.58	1.46 × 10^−3^	
24	O-phospho-L-threonine	258.04	862.43	3.14	0.46	2.03 × 10^−2^	
25	Taurocholate	514.28	278.19	3.26	16.69	1.52 × 10^−2^	Bile acids, alcohols and derivatives
26	Taurochenodeoxycholate	498.29	232.76	3.21	6.00	1.43 × 10^−4^	
27	Taurodeoxycholic acid	517.33	275.10	1.77	4.95	9.16 × 10^−5^	
28	D-ribulose 5-phosphate	213.01	877.22	1.27	0.63	1.10 × 10^−2^	Carbohydrates and carbohydrate conjugates
29	Alpha-D-Glucose	179.06	559.37	5.12	0.61	1.03 × 10^−2^	
30	D-mannose 1-phosphate	259.02	852.54	5.43	0.49	4.68 × 10^−2^	
31	D-glucosamine 6-phosphate	260.05	923.51	3.55	0.47	3.40 × 10^−2^	
32	D-erythrose 4-phosphate	199.00	871.81	1.05	0.46	1.57 × 10^−2^	
33	D-mannose-6-phosphate	225.01	914.63	3.36	0.29	2.33 × 10^−2^	
34	D-glucose 6-phosphate	278.06	912.03	6.78	0.18	4.49 × 10^−3^	
35	Stearic acid	278.06	912.03	6.78	0.18	4.49 × 10^−3^	Fatty acids and conjugates
36	Eicosapentaenoic acid	283.26	185.85	5.71	5.20	1.10 × 10^−2^	
37	Myristic acid	303.23	62.55	1.03	1.35	3.79 × 10^−2^	
38	Adipic acid	227.20	73.21	5.54	0.69	1.15 × 10^−2^	
39	1-oleoyl-sn-glycero-3-phosphocholine	522.36	386.97	7.53	2.32	1.52 × 10^−2^	Glycerophosphocholines
40	1-stearoyl-sn-glycerol 3-phosphocholine	568.34	366.68	1.92	1.97	3.51 × 10^−2^	
41	1-stearoyl-2-hydroxy-sn-glycero-3-phosphocholine	524.37	370.20	2.03	1.63	4.98 × 10^−2^	
42	1,2-dioleoyl-sn-glycero-3-phosphatidylcholine	808.58	256.14	6.31	1.33	3.20 × 10^−2^	

**Table 2:** DE miRNA and targeting genes in co-expression network.

	KEGG ID	Description	miRNA	Target DEGs
1	ko02010	ABC transporters	mmu-miR-3065-3p/oan-miR-1386	K05653 (*LOC101797091*)
2	ko02010	ABC transporters	cja-miR-3065/hsa-miR-3065-3p/mmu-miR-3065-3p/	K05654 (*LOC101797680/* *LOC119718657*)
3	ko05034	Alcoholism	gga-miR-132b-5p	K11254 (*H4*)
4	ko05014/ko05022	Amyotrophic lateral sclerosis/Pathways of neurodegeneration-multiple diseases	ocu-miR-16a-5p	K10408 (*DNAH9*)
5	ko04976/ko04964	Bile secretion/Proximal tubule bicarbonate reclamation	gga-miR-132b-5p	K13575 (*SLC4A4*)
6	ko00260/ko00670	Glycine, serine, and threonine metabolism/One carbon pool by folate	mmu-miR-3065-3p	K00315 (*DMGDH*)
7	ko04931/ko04917	Insulin resistance/Prolactin signaling pathway	oan-miR-1386	K04696 (*SOCS3*)
8	ko04082/ko04080	Neuroactive ligand signaling/Neuroactive ligand–receptor interaction	mmu-miR-199a-3p	K05221 (*P2RX6*)
9	ko04723/ko04080	Retrograde endocannabinoid signaling/Neuroactive ligand–receptor interaction	ggo-miR-148a/hsa-miR-148a-3p/dre-miR-148/ola-miR-148	K05190 (*LOC101798492*)
10	ko04721/ko04082	Synaptic vesicle cycle/Neuroactive ligand signaling	oan-miR-1386	K05038 (*SLC6A9*)

## Data Availability

The raw datasets used and analyzed during the current study are available from the corresponding author upon reasonable request.

## Supplementary Materials

The following supporting information can be downloaded at: https://www.mdpi.com/article/10.3390/ani16060934/s1, Table S1. Nutrient levels of diets at each stage; Table S2. Real-time PCR primers and conditions; Table S3. Statistics of mRNA-seq quality control; Table S4. Differentially expressed mRNA; Table S5. Statistics of miRNA-seq quality control; Table S6. Correlation analysis of miRNA–mRNA; Table S7. Up target DEG_go_enrich (down DE miRNA); Table S8. Down target DEG_go_enrich (up DE miRNA); Table S9. Correlation analysis of DAMs and DEGs; Figure S1. Quality control (QC) of experimental data; Figure S2. Pearson correlation analysis and MCC test of quality control samples.
