# Supplementary material for: Integrated miRNAs, Transcriptome, and Metabolome Uncover Underlying Mechanisms for Breast Muscle Metabolic Regulation in Liancheng White and Cherry Valley Ducks
Source: Animals (Basel). 2026 Mar 16;16(6):934. doi: 10.3390/ani16060934 (PMC13023296; doi:10.3390/ani16060934)
Supplement: Supplementary file 1 [file animals-16-00934-s001.zip › Figure S1. Quality control (QC) of experimental data..pdf]

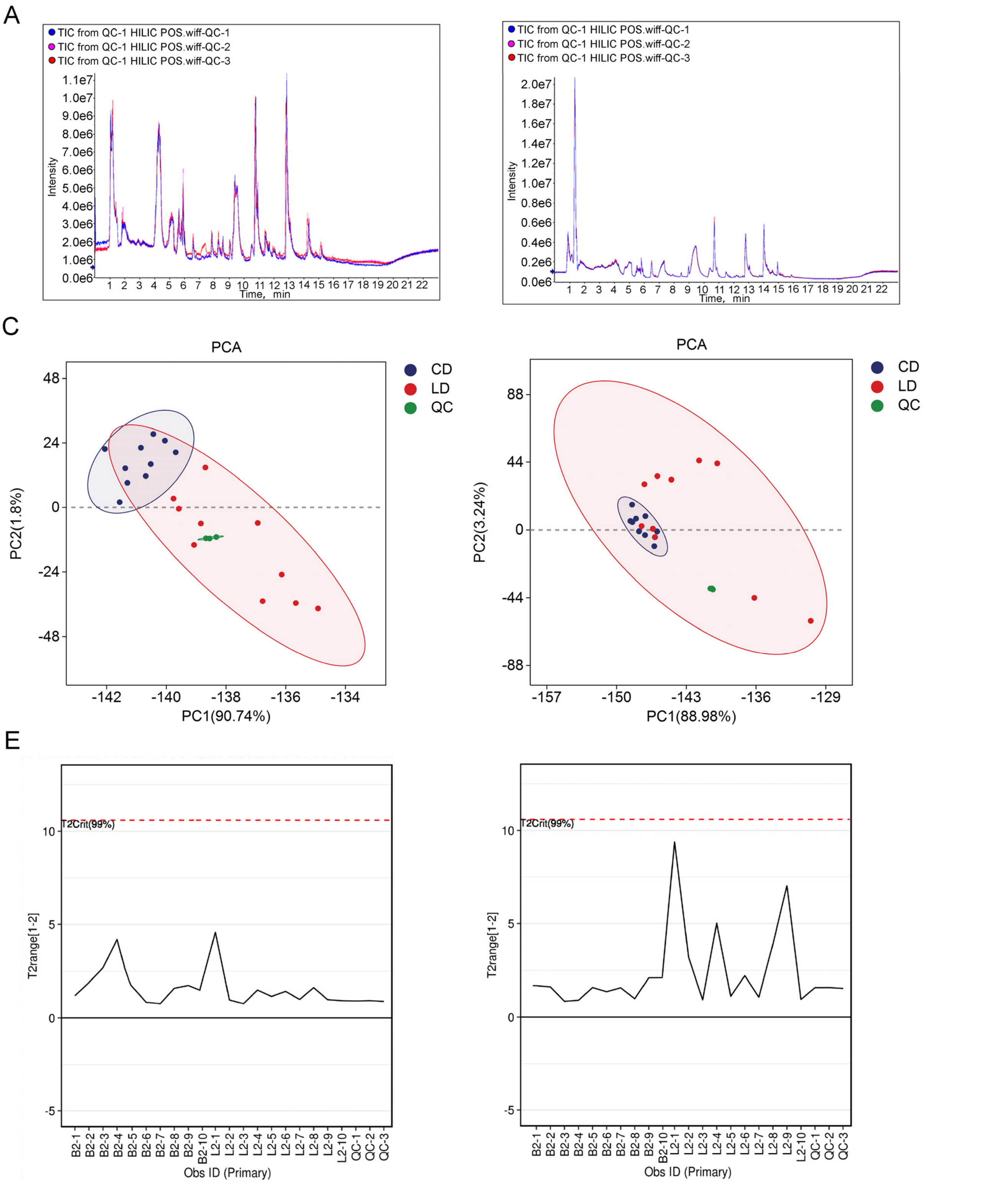

Figure S1. Quality control (QC) of experimental data. (A) Comparison of the total ion chromatogram (TIC) of QC samples in positive ion mode (left) and negative ion mode (right). (B) Principal component analysis (PCA) of the identified metabolites showing QC samples clustered together both in positive ion mode (left) and negative ion mode (right). (C) The Hotellings T2 plot of the samples in the positive (left) and negative (right) ion modes showed that all samples were within the 99 % confidence interval.
