# Supplementary material for: Integrated miRNAs, Transcriptome, and Metabolome Uncover Underlying Mechanisms for Breast Muscle Metabolic Regulation in Liancheng White and Cherry Valley Ducks
Source: Animals (Basel). 2026 Mar 16;16(6):934. doi: 10.3390/ani16060934 (PMC13023296; doi:10.3390/ani16060934)
Supplement: Supplementary file 1 [file animals-16-00934-s001.zip › Figure S2. Pearson correlation analysis and MCC test of quality control samples..pdf]

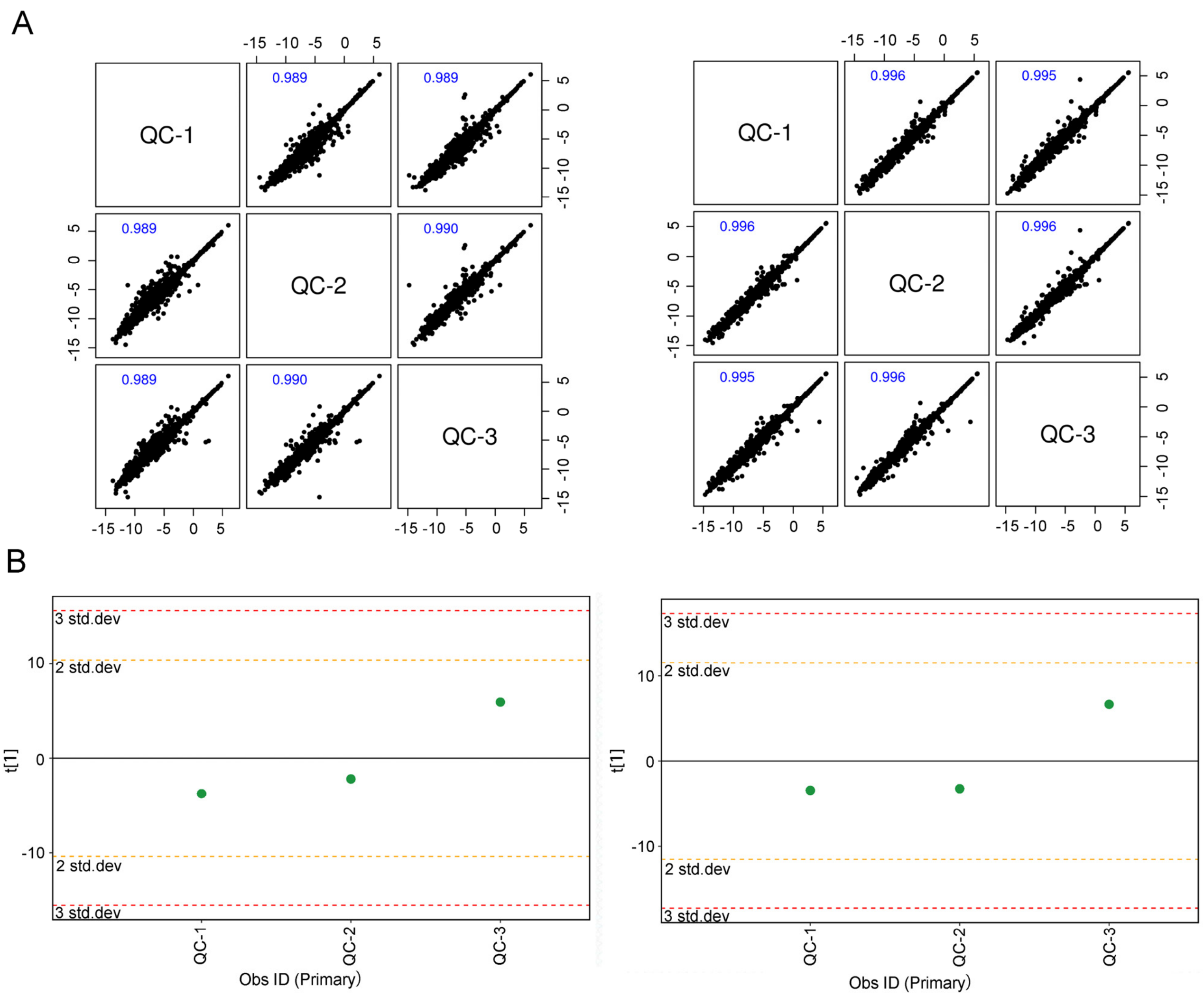

Figure S2. Pearson correlation analysis and MCC test of quality control samples. (A) Pearson correlation analysis on the QC samples in positive ion mode (left) and negative ion mode (right). (B) The MCC test in positive ion mode (left) and negative ion mode (right). MCC test showed that the QC sample had less volatility in the range of positive and negative standard deviations.
