# Supplementary material for: Integrated miRNAs, Transcriptome, and Metabolome Uncover Underlying Mechanisms for Breast Muscle Metabolic Regulation in Liancheng White and Cherry Valley Ducks
Source: Animals (Basel). 2026 Mar 16;16(6):934. doi: 10.3390/ani16060934 (PMC13023296; doi:10.3390/ani16060934)
Supplement: Supplementary file 1 [file animals-16-00934-s001.zip › Table S1. Nutrient levels of diets at each stage.pdf]

Table S1. Nutrient levels of diets at each stage

Nutrient levels / %	0-21 days of age	22-60 days of age	61-130 days of age	131-300 days of age
Crude protein	≥17.5	≥16.0	≥9.0	≥16.0
Crude fiber	≤7.0	≤7.0	≤12.0	≤6.0
Crude ash	≤12.0	≤12.0	≤13.0	≤13.0
Calcium	0.7～1.4	0.7～1.4	0.8～2.5	0.8～1.4
Phosphorus	0.35～1.0	0.35～1.0	0.35～1	0.35～0.75
NaCl	0.25～0.80	0.25～0.80	0.25～0.80	0.30～0.80
Methionine	0.20～0.90	0.20～0.90	0.20～0.90	0.20～0.90
Water	≤13.0	≤13.0	≤13.0	≤13.0
