# Supplementary material for: Integrated miRNAs, Transcriptome, and Metabolome Uncover Underlying Mechanisms for Breast Muscle Metabolic Regulation in Liancheng White and Cherry Valley Ducks
Source: Animals (Basel). 2026 Mar 16;16(6):934. doi: 10.3390/ani16060934 (PMC13023296; doi:10.3390/ani16060934)
Supplement: Supplementary file 1 [file animals-16-00934-s001.zip › Table S2. Real-time PCR primers and conditions.pdf]

**Table S2.** Real-time PCR primers and conditions

| Gene | Genbank accession | Primer sequences (5'to3') | Size (bp) | Annealing (℃) |
|---|---|---|---|---|
| Duck *GAPDH* | XM_005016745.2 | GGAGCTGCCCAGAACATTATC<br>GCAGGTCAGGTCCACGACA | 141 | 60 |
| Duck *CD34* | XM_027444782.2 | GTCCCCAAGCCCATCACA<br>GCAGGTGTTGGACTCGTTGA | 87 | 60 |
| Duck *MYBPH* | XM_027444972.2 | GCTACAACACGCACCTCTTC<br>TGGGGTCTTCCCGTATCTC | 93 | 60 |
| Duck *AKR1D1* | XM_027448117.2 | GTGCCACTTGGGAGGCTATG<br>GGTTGCTGACGGGTTTGTGCTT | 135 | 60 |
| Duck *SOCS3* | XM_005031814.4 | GCCTGAAGACGTTCAGCTCCAA<br>CGTCACCGTGCTCCAGTAGAA | 95 | 60 |
| oan - miR - 1386-F | —— | AACGCGCTCCTGGCTGGCT | —— | 60 |
| U6-F | —— | CTCGCTTCGGCAGCACA | —— | 60 |
