# Supplementary material for: Integrated miRNAs, Transcriptome, and Metabolome Uncover Underlying Mechanisms for Breast Muscle Metabolic Regulation in Liancheng White and Cherry Valley Ducks
Source: Animals (Basel). 2026 Mar 16;16(6):934. doi: 10.3390/ani16060934 (PMC13023296; doi:10.3390/ani16060934)
Supplement: Supplementary file 1 [file animals-16-00934-s001.zip › Table S3. Statistics of mRNA-seq quality control.pdf]

Table S3. Statistics of mRNA-seq quality control

Library	Raw_reads	Raw_ bases	Clean_reads	Clean_ bases	Error_ rate	Q20	Q30	GC_pct%	Total_map
BD1	48565502	7.28G	45617436	6.84G	0.01	98.89	96.8	53.62	36424489(79.85%)
BD2	44576690	6.69G	42617330	6.39G	0.01	98.85	96.7	52.36	33872044(79.48%)
BD3	52182686	7.83G	49030704	7.35G	0.01	98.88	96.79	54.17	37474837(76.43%)
LD1	46171828	6.93G	44692250	6.7G	0.01	98.76	96.49	51.59	36616449(81.93%)
LD2	48323720	7.25G	46122618	6.92G	0.01	98.69	96.34	52.45	37180378(80.61%)
LD3	45203404	6.78G	43456084	6.52G	0.01	98.7	96.31	52.25	34426884(79.22%)
