# Supplementary material for: Integrated miRNAs, Transcriptome, and Metabolome Uncover Underlying Mechanisms for Breast Muscle Metabolic Regulation in Liancheng White and Cherry Valley Ducks
Source: Animals (Basel). 2026 Mar 16;16(6):934. doi: 10.3390/ani16060934 (PMC13023296; doi:10.3390/ani16060934)
Supplement: Supplementary file 1 [file animals-16-00934-s001.zip › Table S4. Differentially expressed mRNA.pdf]

Table S4. Differentially expressed mRNA.

Gene_name	Gene_ID	log Fold Change	P value	FDR
LOC119715258	119715258	-7.16	1.15×10^{-39}	1.30×10^{-35}
LOC113842285	113842285	-7.64	2.37×10^{-36}	1.35×10^{-32}
LOC119718661	119718661	-10.17	3.69×10^{-28}	$1.40 \times 10\text{-}^{24}$
LOC101801612	101801612	-7.31	4.63×10^{-27}	1.32×10^{-23}
LOC110354782	110354782	5.10	2.14×10^{-21}	4.86×10^{-18}
NPM3	101798086	4.73	8.59×10^{-19}	1.54×10^{-15}
LOC119715450	119715450	-4.18	9.49×10^{-19}	1.54×10^{-15}
LOC119713066	119713066	-7.08	3.10×10^{-18}	4.40×10^{-15}
PCP4	101796342	-4.62	7.62×10^{-18}	9.62×10^{-15}
LOC113841252	113841252	-4.37	2.69×10^{-17}	3.06×10^{-14}
LOC119713150	119713150	-3.91	3.05×10^{-17}	3.15×10^{-14}
LOC119717843	119717843	-3.98	9.73×10^{-17}	9.21×10^{-14}
LOC119713237	119713237	4.02	4.34×10^{-16}	3.79×10^{-13}
AMPD3	101803104	3.72	5.04×10^{-16}	4.09×10^{-13}
LOC119718653	119718653	-4.24	2.78×10^{-15}	2.11×10^{-12}
LOC113845410	113845410	4.31	3.12×10^{-15}	2.21×10^{-12}
LOC101792948	101792948	3.81	3.49×10^{-15}	2.33×10^{-12}
P2RX6	101790078	-4.18	5.04×10^{-15}	3.18×10^{-12}
LOC119715393	119715393	-4.13	4.36×10^{-14}	2.60×10^{-11}
SLC25A25	101790469	3.40	5.20×10^{-14}	2.95×10^{-11}
AP3B2	101791839	-3.76	6.09×10^{-14}	3.29×10^{-11}
LOC110354783	110354783	4.28	1.10×10^{-13}	5.69×10^{-11}
LOC113845374	113845374	-5.99	4.39×10^{-13}	2.17×10^{-10}
LOC119713152	119713152	3.91	8.14×10^{-13}	3.85×10^{-10}
EGR3	119713540	3.67	9.32×10^{-13}	4.07×10^{-10}
LOC119715623	119715623	3.79	9.33×10^{-13}	4.07×10^{-10}
LOC113841786	113841786	-3.16	1.91×10^{-12}	8.05×10^{-10}
MYBPH	101804684	3.06	3.81×10^{-12}	1.55×10^{-9}
LOC119717610	119717610	3.11	6.08×10^{-12}	2.38×10^{-9}
INHBA	101795448	3.06	7.07×10^{-12}	2.67×10^{-9}
LOC106014873	106014873	-3.20	8.42×10^{-12}	3.08×10^{-9}
LOC119713068	119713068	-4.83	1.49×10^{-11}	5.28×10^{-9}
LOC101793739	101793739	-3.69	1.56×10^{-11}	5.37×10^{-9}
ST13	101796439	3.07	1.64×10^{-11}	5.47×10^{-9}
PM20D2	101799838	2.91	3.13×10^{-11}	1.02×10^{-8}
LOC113843893	113843893	-3.85	4.11×10^{-11}	1.30×10^{-8}
CD34	101804360	-2.88	1.10×10^{-10}	3.38×10^{-8}
EGR1	101800060	2.82	1.17×10^{-10}	3.50×10^{-8}
LOC119713166	119713166	2.86	1.54×10^{-10}	4.48×10^{-8}
FOS	101801698	2.78	1.80×10^{-10}	5.10×10^{-8}
PTPRT	101803878	2.84	3.32×10^{-10}	9.02×10^{-8}

RASL10B	101797335	-2.76	3.34×10^{-10}	9.02×10^{-8}
LOC101800810	101800810	3.03	3.66×10^{-10}	9.67×10^{-8}
LOC119715059	119715059	-4.19	5.46×10^{-10}	1.41×10^{-7}
LOC119718672	119718672	3.60	6.45×10^{-10}	1.63×10^{-7}
H4	119713606	3.50	1.12×10^{-9}	2.77×10^{-7}
LOC113845379	113845379	-2.70	1.30×10^{-9}	3.15×10^{-7}
ADAMTS8	101799556	2.72	1.44×10^{-9}	3.40×10^{-7}
LOC119712991	119712991	-3.08	1.87×10^{-9}	4.33×10^{-7}
LOC119717358	119717358	2.96	2.05×10^{-9}	4.65×10^{-7}
LOC101791228	101791228	3.17	2.61×10^{-9}	5.82×10^{-7}
BLOC1S1	119714706	-3.29	2.71×10^{-9}	5.92×10^{-7}
LOC106019355	106019355	-2.69	4.87×10^{-9}	1.04×10^{-6}
LOC106015748	106015748	3.11	5.23×10^{-9}	1.10×10^{-6}
LRRC56	101795329	-3.05	5.55×10^{-9}	1.15×10^{-6}
LOC119718656	119718656	2.78	7.20×10^{-9}	1.46×10^{-6}
CSRNP1	101792404	2.55	7.33×10^{-9}	1.46×10^{-6}
LOC119713077	119713077	2.63	9.38×10^{-9}	1.84×10^{-6}
BTG2	101793601	2.45	1.22×10^{-8}	2.36×10^{-6}
LOC110351955	110351955	2.96	1.27×10^{-8}	2.39×10^{-6}
KCNN1	119714187	-2.53	1.34×10^{-8}	2.50×10^{-6}
LOC113845392	113845392	-3.78	1.50×10^{-8}	2.74×10^{-6}
MFSD2A	119713663	2.86	1.62×10^{-8}	2.92×10^{-6}
CA12	101795734	2.59	2.02×10^{-8}	3.58×10^{-6}
RERGL	101802098	-2.59	2.24×10^{-8}	3.91×10^{-6}
LOC119713059	119713059	3.29	2.38×10^{-8}	4.09×10^{-6}
LOC101792067	101792067	2.41	3.16×10^{-8}	5.35×10^{-6}
DRP2	101801801	2.54	4.14×10^{-8}	6.91×10^{-6}
LOC119714453	119714453	-3.07	7.77×10^{-8}	1.28×10^{-5}
LOC119715617	119715617	2.34	9.19×10^{-8}	1.49×10^{-5}
LOC113845068	113845068	2.37	1.04×10^{-7}	1.66×10^{-5}
LOC106019907	106019907	-2.98	1.17×10^{-7}	1.85×10^{-5}
LOC119712994	119712994	2.28	1.22×10^{-7}	1.90×10^{-5}
C7	101800101	-2.36	1.39×10^{-7}	2.11×10^{-5}
PRSS12	101798435	-2.81	1.39×10^{-7}	2.11×10^{-5}
LOC110351820	110351820	-2.90	1.45×10^{-7}	2.16×10^{-5}
TINAG	101794402	2.53	1.48×10^{-7}	2.19×10^{-5}
ADAMTS20	101801774	2.31	1.59×10^{-7}	2.32×10^{-5}
FADS6	101800995	-2.67	1.72×10^{-7}	2.47×10^{-5}
LOC119717827	119717827	2.55	2.21×10^{-7}	3.13×10^{-5}
LOC113841196	113841196	-2.53	2.32×10^{-7}	3.25×10^{-5}
CLHC1	101802369	-2.94	2.38×10^{-7}	3.27×10^{-5}
RASD1	101801185	2.36	2.39×10^{-7}	3.27×10^{-5}
LOC119716536	119716536	2.34	2.51×10^{-7}	3.40×10^{-5}
MYC	101803370	2.22	2.68×10^{-7}	3.56×10^{-5}
SNCA	101794489	2.83	2.70×10^{-7}	3.56×10^{-5}

LOC119718657	119718657	2.65	2.92×10^{-7}	3.81×10^{-5}
ACOT11	101801459	2.23	2.97×10^{-7}	3.83×10^{-5}
TMEM108	101792079	-2.32	3.16×10^{-7}	4.02×10^{-5}
EVA1B	113839786	-2.38	3.19×10^{-7}	4.02×10^{-5}
VIT	101797536	3.75	5.22×10^{-7}	6.52×10^{-5}
LOC101794508	101794508	2.13	5.37×10^{-7}	6.63×10^{-5}
LOC101796082	101796082	-3.27	5.67×10^{-7}	6.93×10^{-5}
SOCS3	101791200	2.32	5.76×10^{-7}	6.96×10^{-5}
VASH2	101804871	2.21	5.88×10^{-7}	7.03×10^{-5}
RBFOX1	101803943	2.21	7.02×10^{-7}	8.31×10^{-5}
SIK1	101804342	2.11	7.39×10^{-7}	8.66×10^{-5}
ASB9	101791098	2.31	7.77×10^{-7}	9.00×10^{-5}
TMEM269	101792812	2.32	8.09×10^{-7}	9.28×10^{-5}
F3	101793475	2.32	8.34×10^{-7}	9.47×10^{-5}
TRIM71	101796941	2.51	1.20×10^{-6}	1.35×10^{-4}
ADAMTSL2	101802351	2.14	1.28×10^{-6}	1.42×10^{-4}
LOC113840177	113840177	-2.19	1.38×10^{-6}	1.52×10^{-4}
CCN1	101799001	2.04	1.56×10^{-6}	1.70×10^{-4}
CNTNAP5	101800307	-2.98	1.60×10^{-6}	1.73×10^{-4}
BBIP1	106015390	-2.24	1.65×10^{-6}	1.77×10^{-4}
CD44	101797405	2.08	1.67×10^{-6}	1.77×10^{-4}
VGLL2	110352167	-2.05	1.79×10^{-6}	1.88×10^{-4}
SNAI3	119718136	-2.20	1.81×10^{-6}	1.89×10^{-4}
PLIN1	101795741	-2.19	1.88×10^{-6}	1.94×10^{-4}
LOC101804899	101804899	2.04	2.05×10^{-6}	2.08×10^{-4}
LOC101794146	101794146	-2.78	2.05×10^{-6}	2.08×10^{-4}
DNAJA4	101801226	2.01	2.13×10^{-6}	2.14×10^{-4}
FAM131B	101794419	2.18	2.16×10^{-6}	2.15×10^{-4}
LOC113843579	113843579	2.55	2.36×10^{-6}	2.33×10^{-4}
LOC101797798	101797798	-2.31	2.59×10^{-6}	2.53×10^{-4}
LOC113840668	113840668	-2.25	2.61×10^{-6}	2.53×10^{-4}
NR4A2	101801493	2.13	2.84×10^{-6}	2.74×10^{-4}
LOC101798492	101798492	2.86	2.89×10^{-6}	2.74×10^{-4}
TMEM150C	101790646	-2.00	2.90×10^{-6}	2.74×10^{-4}
LOC113843146	113843146	-2.18	3.16×10^{-6}	2.96×10^{-4}
LOC119715006	119715006	-2.04	3.21×10^{-6}	2.99×10^{-4}
SORL1	101803246	-2.14	3.57×10^{-6}	3.30×10^{-4}
TNFRSF6B	101801227	2.25	3.80×10^{-6}	3.45×10^{-4}
GLIS3	101803509	2.04	3.81×10^{-6}	3.45×10^{-4}
HSPA8	101801738	1.95	3.83×10^{-6}	3.45×10^{-4}
CUBN	101794727	2.26	4.04×10^{-6}	3.61×10^{-4}
LOC101797243	101797243	2.81	4.17×10^{-6}	3.70×10^{-4}
LOC101804756	101804756	2.01	4.46×10^{-6}	3.88×10^{-4}
LOC113843518	113843518	-2.20	4.46×10^{-6}	3.88×10^{-4}
LOC106018567	106018567	-2.30	4.47×10^{-6}	3.88×10^{-4}

MTMR7	101805213	2.39	4.66×10^{-6}	4.01×10^{-4}
GABRB3	101795544	-2.03	4.84×10^{-6}	4.13×10^{-4}
GATM	101796257	1.95	4.97×10^{-6}	4.21×10^{-4}
LIPG	101799752	2.29	5.20×10^{-6}	4.37×10^{-4}
LOC113840518	113840518	-1.93	5.45×10^{-6}	4.55×10^{-4}
LOC106020235	106020235	-1.92	5.95×10^{-6}	4.90×10^{-4}
PLXNC1	101790206	-1.97	5.96×10^{-6}	4.90×10^{-4}
CTSL	101803168	1.91	6.24×10^{-6}	5.10×10^{-4}
SPSB4	101789728	2.11	6.43×10^{-6}	5.21×10^{-4}
NYX	101805440	1.94	6.86×10^{-6}	5.53×10^{-4}
LOC110352112	110352112	2.35	6.96×10^{-6}	5.57×10^{-4}
LOC113843458	113843458	-2.03	7.28×10^{-6}	5.78×10^{-4}
VSTM2B	113844984	-2.19	7.44×10^{-6}	5.82×10^{-4}
ITGB8	101797604	2.19	7.44×10^{-6}	5.82×10^{-4}
STC2	101801672	1.93	7.81×10^{-6}	6.07×10^{-4}
DENND2D	101799913	2.16	8.54×10^{-6}	6.60×10^{-4}
THBS1	101793322	1.89	9.64×10^{-6}	7.39×10^{-4}
TMPRSS9	101791526	2.25	1.02×10^{-5}	7.79×10^{-4}
GCLC	101799767	-2.18	1.10×10^{-5}	8.30×10^{-4}
ZMAT4	101804593	-2.28	1.13×10^{-5}	8.48×10^{-4}
LOC110354662	110354662	2.45	1.13×10^{-5}	8.48×10^{-4}
HSPH1	101798550	1.86	1.14×10^{-5}	8.49×10^{-4}
FKBP5	101803184	1.85	1.25×10^{-5}	9.19×10^{-4}
LOC113843797	113843797	2.30	1.25×10^{-5}	9.19×10^{-4}
FN1	101800175	-1.85	1.33×10^{-5}	9.71×10^{-4}
GAS2L2	101797145	-2.54	1.38×10^{-5}	9.98×10^{-4}
TTLL9	101795946	-1.96	1.41×10^{-5}	1.01×10^{-3}
CACNB4	101798353	-1.94	1.42×10^{-5}	1.01×10^{-3}
PIEZO2	101804683	-2.18	1.42×10^{-5}	1.01×10^{-3}
KY	101793587	1.87	1.50×10^{-5}	1.06×10^{-3}
LOC113845380	113845380	-1.85	1.52×10^{-5}	1.06×10^{-3}
LOC119713003	119713003	-2.40	1.63×10^{-5}	1.14×10^{-3}
TNFRSF11B	101791841	1.84	1.66×10^{-5}	1.15×10^{-3}
MTLN	119716547	-1.97	1.66×10^{-5}	1.15×10^{-3}
LOC113845399	113845399	-1.83	1.69×10^{-5}	1.16×10^{-3}
LOC106018381	106018381	-2.96	1.78×10^{-5}	1.20×10^{-3}
LOC119717614	119717614	-1.87	1.78×10^{-5}	1.20×10^{-3}
FBXO30	101803240	1.81	1.82×10^{-5}	1.23×10^{-3}
LOC119717136	119717136	2.69	1.90×10^{-5}	1.27×10^{-3}
TPMT	101797003	-1.87	1.91×10^{-5}	1.27×10^{-3}
LOC113843097	113843097	2.04	2.26×10^{-5}	1.49×10^{-3}
SNX16	101792242	1.79	2.34×10^{-5}	1.53×10^{-3}
UCHL1	101799480	1.80	2.35×10^{-5}	1.53×10^{-3}
SERPIND1	101798143	-1.91	2.36×10^{-5}	1.53×10^{-3}
LOC113840577	113840577	-2.04	2.43×10^{-5}	1.57×10^{-3}

CCDC180	101791450	1.90	2.50×10^{-5}	1.60×10^{-3}
SYT3	119715455	-2.35	2.53×10^{-5}	1.61×10^{-3}
FAM13A	101796615	1.80	2.78×10^{-5}	1.76×10^{-3}
HES1	113844479	1.79	2.89×10^{-5}	1.82×10^{-3}
LOC101796443	101796443	2.02	2.93×10^{-5}	1.84×10^{-3}
ZNF469	101804975	2.05	2.96×10^{-5}	1.84×10^{-3}
LOC113841624	113841624	2.13	3.20×10^{-5}	1.99×10^{-3}
PKIG	101796681	1.81	3.28×10^{-5}	2.02×10^{-3}
LOC113842967	113842967	1.79	3.29×10^{-5}	2.02×10^{-3}
TNC	101804896	1.77	3.38×10^{-5}	2.06×10^{-3}
FLVCR1	101790978	1.76	3.49×10^{-5}	2.12×10^{-3}
KBTBD3	101792953	2.29	3.69×10^{-5}	2.23×10^{-3}
LOC101803486	101803486	-1.75	3.78×10^{-5}	2.27×10^{-3}
LOC101793418	101793418	-1.72	4.01×10^{-5}	2.39×10^{-3}
LOC113845662	113845662	2.03	4.03×10^{-5}	2.39×10^{-3}
MIDN	119714188	1.77	4.04×10^{-5}	2.39×10^{-3}
LOC119715208	119715208	1.78	4.09×10^{-5}	2.41×10^{-3}
LOC101802067	101802067	-2.00	4.14×10^{-5}	2.42×10^{-3}
MYOZ2	101793698	1.94	4.20×10^{-5}	2.45×10^{-3}
TTPA	101790502	-1.76	4.27×10^{-5}	2.47×10^{-3}
SLC4A4	101798600	1.74	4.28×10^{-5}	2.47×10^{-3}
RASGRF1	101789884	-1.90	4.35×10^{-5}	2.50×10^{-3}
SLC6A9	119717606	1.98	4.49×10^{-5}	2.56×10^{-3}
LOC119715156	119715156	1.83	4.59×10^{-5}	2.61×10^{-3}
LOC106020121	106020121	1.74	4.65×10^{-5}	2.63×10^{-3}
LOC101790845	101790845	-1.88	4.71×10^{-5}	2.65×10^{-3}
HOXD3	106015148	-2.16	4.90×10^{-5}	2.74×10^{-3}
OPLAH	119716150	-1.80	5.04×10^{-5}	2.80×10^{-3}
LOC113840607	113840607	-2.04	5.06×10^{-5}	2.80×10^{-3}
PAK1	101795474	1.93	5.41×10^{-5}	2.98×10^{-3}
APOLD1	101798726	1.70	5.54×10^{-5}	3.04×10^{-3}
LOC113845286	113845286	1.83	5.65×10^{-5}	3.08×10^{-3}
LOC113843088	113843088	-1.92	5.92×10^{-5}	3.22×10^{-3}
FUNDC2	101797055	-1.68	6.23×10^{-5}	3.36×10^{-3}
LOC119715889	119715889	2.05	6.24×10^{-5}	3.36×10^{-3}
MCOLN3	101794809	1.93	6.37×10^{-5}	3.41×10^{-3}
LOC101805353	101805353	2.00	6.39×10^{-5}	3.41×10^{-3}
CNTFR	101790239	1.85	6.59×10^{-5}	3.50×10^{-3}
C1H12orf57	113842424	-1.69	6.63×10^{-5}	3.50×10^{-3}
BAIAP2L1	101804329	-1.90	6.95×10^{-5}	3.65×10^{-3}
LOC101792484	101792484	-1.66	7.00×10^{-5}	3.67×10^{-3}
RPUSD3	119718222	-1.67	7.23×10^{-5}	3.77×10^{-3}
LOC119714045	119714045	-2.10	7.41×10^{-5}	3.84×10^{-3}
LOC119714426	119714426	2.61	7.87×10^{-5}	4.06×10^{-3}
ATP6V0E2	113843982	1.79	7.94×10^{-5}	4.08×10^{-3}

LOC101797091	101797091	-1.69	8.29×10^{-5}	4.24×10^{-3}
LOC101797147	101797147	-1.91	8.79×10^{-5}	4.48×10^{-3}
LOC119713017	119713017	-2.24	9.04×10^{-5}	4.58×10^{-3}
LOC101796643	101796643	-1.68	9.08×10^{-5}	4.58×10^{-3}
TENT5B	101800366	1.64	9.21×10^{-5}	4.63×10^{-3}
CDC42EP1	110352046	-1.65	9.35×10^{-5}	4.68×10^{-3}
MB	101804689	-1.63	9.42×10^{-5}	4.69×10^{-3}
LOC119714288	119714288	-2.12	9.52×10^{-5}	4.71×10^{-3}
LOC101796889	101796889	1.69	9.54×10^{-5}	4.71×10^{-3}
LOC101796631	101796631	-1.79	9.68×10^{-5}	4.76×10^{-3}
DMGDH	101802594	-1.73	9.83×10^{-5}	4.81×10^{-3}
DPP7	101791397	1.65	9.88×10^{-5}	4.82×10^{-3}
FMOD	101789778	-1.82	1.00×10^{-4}	4.85×10^{-3}
DGLUCY	101791243	-1.72	1.01×10^{-4}	4.86×10^{-3}
PIMREG	101795463	1.66	1.03×10^{-4}	4.96×10^{-3}
LOC113839855	113839855	2.17	1.04×10^{-4}	4.98×10^{-3}
PRKG2	101790644	2.51	1.07×10^{-4}	5.10×10^{-3}
TMEM86A	101802063	1.63	1.14×10^{-4}	5.41×10^{-3}
TRIM63	119713599	1.61	1.17×10^{-4}	5.54×10^{-3}
ALDH1A3	101793139	1.69	1.19×10^{-4}	5.57×10^{-3}
LOC119716356	119716356	-1.67	1.19×10^{-4}	5.57×10^{-3}
ZYX	101794000	1.63	1.20×10^{-4}	5.60×10^{-3}
LOC110351385	110351385	1.65	1.21×10^{-4}	5.65×10^{-3}
RGCC	101803833	1.63	1.22×10^{-4}	5.66×10^{-3}
NGEF	101799143	1.61	1.27×10^{-4}	5.87×10^{-3}
PRRT1B	113845478	-2.14	1.28×10^{-4}	5.87×10^{-3}
CCDC92B	101790736	-1.72	1.30×10^{-4}	5.97×10^{-3}
NR4A1	101800531	1.60	1.32×10^{-4}	6.02×10^{-3}
HIGD1A	110353390	1.60	1.33×10^{-4}	6.05×10^{-3}
LOC101800293	101800293	1.66	1.34×10^{-4}	6.08×10^{-3}
CHORDC1	101791022	1.65	1.35×10^{-4}	6.08×10^{-3}
NT5DC1	101804281	-1.69	1.38×10^{-4}	6.19×10^{-3}
LOC113842963	113842963	-2.14	1.39×10^{-4}	6.23×10^{-3}
NR4A3	101795028	1.61	1.40×10^{-4}	6.26×10^{-3}
LOC110353849	110353849	1.99	1.41×10^{-4}	6.27×10^{-3}
LOC101805082	101805082	1.61	1.43×10^{-4}	6.32×10^{-3}
LOC106016400	106016400	1.69	1.45×10^{-4}	6.38×10^{-3}
CPXM2	101793162	-2.14	1.46×10^{-4}	6.38×10^{-3}
KCNC2	101802817	-1.73	1.47×10^{-4}	6.39×10^{-3}
LOC101799154	101799154	-1.62	1.47×10^{-4}	6.39×10^{-3}
LOC101791306	101791306	1.94	1.48×10^{-4}	6.43×10^{-3}
NAAA	101804788	1.60	1.52×10^{-4}	6.54×10^{-3}
MCM3	101797341	1.86	1.52×10^{-4}	6.54×10^{-3}
OTUD3	101793885	-1.62	1.59×10^{-4}	6.79×10^{-3}
ARHGAP19	101798934	2.18	1.61×10^{-4}	6.87×10^{-3}

LOC119712983	119712983	1.91	1.63×10^{-4}	6.94×10^{-3}
FAM162B	113843253	-1.73	1.64×10^{-4}	6.96×10^{-3}
LOC106018147	106018147	1.80	1.66×10^{-4}	6.98×10^{-3}
LOC119715240	119715240	1.82	1.66×10^{-4}	6.98×10^{-3}
LOC113839874	113839874	-1.61	1.77×10^{-4}	7.43×10^{-3}
GRID1	101800190	1.96	1.81×10^{-4}	7.52×10^{-3}
DNAH9	101790717	-1.68	1.81×10^{-4}	7.52×10^{-3}
LOC119714271	119714271	1.60	1.94×10^{-4}	8.05×10^{-3}
LOC110352460	110352460	-2.08	1.96×10^{-4}	8.10×10^{-3}
LOC119717645	119717645	2.03	2.02×10^{-4}	8.31×10^{-3}
SLC7A6	101797183	1.58	2.03×10^{-4}	8.31×10^{-3}
ASNS	101797455	1.57	2.03×10^{-4}	8.31×10^{-3}
LOC113842798	113842798	2.86	2.04×10^{-4}	8.31×10^{-3}
LOC101795284	101795284	1.60	2.08×10^{-4}	8.44×10^{-3}
LOC101797129	101797129	1.81	2.14×10^{-4}	8.65×10^{-3}
LOC101803804	101803804	-2.34	2.18×10^{-4}	8.78×10^{-3}
TM4SF18	101796112	-1.71	2.20×10^{-4}	8.82×10^{-3}
LOC119717907	119717907	1.73	2.21×10^{-4}	8.83×10^{-3}
RFLNA	101798185	1.76	2.24×10^{-4}	8.92×10^{-3}
KLF4	113840217	1.61	2.26×10^{-4}	8.97×10^{-3}
CCDC134	101792476	1.56	2.38×10^{-4}	9.42×10^{-3}
SYNC	119713666	1.63	2.42×10^{-4}	9.56×10^{-3}
LOC119717883	119717883	1.88	2.47×10^{-4}	9.71×10^{-3}
DKK2	101790422	1.55	2.50×10^{-4}	9.79×10^{-3}
LOC101792201	101792201	-1.85	2.52×10^{-4}	9.85×10^{-3}
TCTA	119718197	-1.58	2.54×10^{-4}	9.86×10^{-3}
KIF16B	101803768	1.54	2.56×10^{-4}	9.86×10^{-3}
KDF1	101799048	-2.43	2.56×10^{-4}	9.86×10^{-3}
LOC101797256	101797256	2.26	2.56×10^{-4}	9.86×10^{-3}
CH25H	101794901	1.96	2.63×10^{-4}	1.01×10^{-2}
LOC110353790	110353790	-2.03	2.66×10^{-4}	1.02×10^{-2}
LOC101794339	101794339	-1.99	2.69×10^{-4}	1.03×10^{-2}
HSPA2	101797658	1.52	2.71×10^{-4}	1.03×10^{-2}
TLR3	101790771	1.86	2.72×10^{-4}	1.03×10^{-2}
NXNL1	101789474	-1.85	2.72×10^{-4}	1.03×10^{-2}
KHDRBS3	101789811	1.53	2.75×10^{-4}	1.03×10^{-2}
COPZ2	119714170	-1.68	2.76×10^{-4}	1.03×10^{-2}
RABEPK	101789781	1.56	2.79×10^{-4}	1.04×10^{-2}
PLBD1	101790786	-1.60	2.81×10^{-4}	1.05×10^{-2}
MSANTD4	101793278	1.62	2.82×10^{-4}	1.05×10^{-2}
LOC113844315	113844315	-1.60	2.83×10^{-4}	1.05×10^{-2}
LMNB2	101791346	1.55	2.92×10^{-4}	1.08×10^{-2}
SHMT1	101802281	1.84	2.94×10^{-4}	1.08×10^{-2}
LOC113842315	113842315	-1.53	2.96×10^{-4}	1.09×10^{-2}
LOC113844122	113844122	-1.98	2.97×10^{-4}	1.09×10^{-2}

PLXND1	101797337	-1.51	3.01×10^{-4}	1.09×10^{-2}
LOC119718652	119718652	-1.80	3.02×10^{-4}	1.09×10^{-2}
DGAT2	101801215	1.52	3.03×10^{-4}	1.10×10^{-2}
LOC113840411	113840411	-2.31	3.04×10^{-4}	1.10×10^{-2}
TLL1	101794710	-1.79	3.20×10^{-4}	1.15×10^{-2}
LOC101801625	101801625	1.52	3.24×10^{-4}	1.16×10^{-2}
IQSEC3	101793376	1.88	3.24×10^{-4}	1.16×10^{-2}
LOC119717600	119717600	1.57	3.38×10^{-4}	1.20×10^{-2}
LOC101801299	101801299	1.51	3.54×10^{-4}	1.26×10^{-2}
LOC119716128	119716128	1.67	3.55×10^{-4}	1.26×10^{-2}
FAM155B	119717870	-1.50	3.58×10^{-4}	1.26×10^{-2}
EXTL1	119713596	-1.49	3.61×10^{-4}	1.27×10^{-2}
LOC101804291	101804291	-1.64	3.63×10^{-4}	1.27×10^{-2}
TENM2	101800778	1.70	3.71×10^{-4}	1.30×10^{-2}
HSP90AA1	101800442	1.48	3.74×10^{-4}	1.30×10^{-2}
CD74	101803352	-1.49	3.74×10^{-4}	1.30×10^{-2}
CHGB	101795000	1.50	3.86×10^{-4}	1.34×10^{-2}
LOC106019797	106019797	-1.50	3.92×10^{-4}	1.35×10^{-2}
SLC27A3	119713836	-1.52	4.05×10^{-4}	1.40×10^{-2}
CDKN1C	113843791	-1.83	4.11×10^{-4}	1.41×10^{-2}
SLC2A3	101791516	1.48	4.16×10^{-4}	1.42×10^{-2}
LOC113845026	113845026	-1.82	4.25×10^{-4}	1.45×10^{-2}
LRRC66	101791452	1.73	4.34×10^{-4}	1.48×10^{-2}
LOC113845389	113845389	-1.98	4.42×10^{-4}	1.50×10^{-2}
LHX1	101795254	1.96	4.48×10^{-4}	1.51×10^{-2}
EFHD1	101800527	1.48	4.51×10^{-4}	1.52×10^{-2}
PPDPF	101794825	-1.46	4.53×10^{-4}	1.52×10^{-2}
GLDN	101790754	1.94	4.58×10^{-4}	1.54×10^{-2}
ZNF385D	101790304	1.55	4.61×10^{-4}	1.54×10^{-2}
AKR1D1	101802488	1.54	4.64×10^{-4}	1.54×10^{-2}
LOC101795858	101795858	-1.59	4.65×10^{-4}	1.54×10^{-2}
BCAS1	101797212	1.95	4.69×10^{-4}	1.55×10^{-2}
LOC101794040	101794040	1.54	4.71×10^{-4}	1.55×10^{-2}
TRABD2B	101795488	1.84	4.74×10^{-4}	1.56×10^{-2}
KIF26A	101790443	-1.48	4.77×10^{-4}	1.56×10^{-2}
QRICH2	113845527	-1.77	5.27×10^{-4}	1.73×10^{-2}
PRUNE2	101797350	-1.86	5.36×10^{-4}	1.75×10^{-2}
FGF12	101798106	1.66	5.37×10^{-4}	1.75×10^{-2}
CHAC1	101792211	1.44	5.56×10^{-4}	1.80×10^{-2}
C4H4orf48	101793824	-1.80	5.57×10^{-4}	1.80×10^{-2}
LOC119713438	119713438	1.66	5.59×10^{-4}	1.80×10^{-2}
CCDC85A	101801575	-1.64	5.63×10^{-4}	1.81×10^{-2}
LOC119718226	119718226	1.43	5.86×10^{-4}	1.87×10^{-2}
ITGB6	101796767	1.44	5.86×10^{-4}	1.87×10^{-2}
DNASE1L3	101796391	-1.44	5.87×10^{-4}	1.87×10^{-2}

RSPH1	101800088	-1.58	5.87×10^{-4}	1.87×10^{-2}
LOC101798048	101798048	-1.45	5.94×10^{-4}	1.88×10^{-2}
FAM53B	101793670	-1.53	6.15×10^{-4}	1.94×10^{-2}
TAF8	119713917	1.74	6.17×10^{-4}	1.94×10^{-2}
NMRK2	101792444	1.44	6.18×10^{-4}	1.94×10^{-2}
LOC101799374	101799374	-1.66	6.23×10^{-4}	1.95×10^{-2}
HK2	119713467	1.43	6.23×10^{-4}	1.95×10^{-2}
FHL2	101803397	-1.78	6.36×10^{-4}	1.98×10^{-2}
NFKBIZ	101792607	1.47	6.39×10^{-4}	1.99×10^{-2}
BMPR1B	101794439	2.07	6.57×10^{-4}	2.04×10^{-2}
LOC119713615	119713615	-1.56	6.67×10^{-4}	2.06×10^{-2}
LOC110352081	110352081	1.94	6.71×10^{-4}	2.07×10^{-2}
DAPK2	101802579	-1.42	6.83×10^{-4}	2.10×10^{-2}
MPP4	101795206	1.63	7.06×10^{-4}	2.17×10^{-2}
RUBCNL	101789675	-1.57	7.08×10^{-4}	2.17×10^{-2}
LOC106019881	106019881	-1.51	7.14×10^{-4}	2.18×10^{-2}
CCDC92	101798738	1.52	7.15×10^{-4}	2.18×10^{-2}
FRMD1	101790727	1.90	7.22×10^{-4}	2.19×10^{-2}
SOX9	101796646	1.84	7.51×10^{-4}	2.27×10^{-2}
LOC101794631	101794631	1.56	7.64×10^{-4}	2.31×10^{-2}
ND3	5405822	-1.40	7.98×10^{-4}	2.41×10^{-2}
MST1R	101805281	1.62	8.04×10^{-4}	2.42×10^{-2}
LOC101804850	101804850	-1.62	8.07×10^{-4}	2.42×10^{-2}
PRKN	101796091	-1.45	8.11×10^{-4}	2.42×10^{-2}
CEBPD	113842846	1.39	8.23×10^{-4}	2.45×10^{-2}
LOC101792819	101792819	-1.40	8.24×10^{-4}	2.45×10^{-2}
RPH3AL	101804495	-1.48	8.36×10^{-4}	2.48×10^{-2}
CCDC13	101792262	-1.46	8.43×10^{-4}	2.49×10^{-2}
PIGX	101798769	1.40	8.59×10^{-4}	2.53×10^{-2}
NUDT6	101798244	-1.64	8.64×10^{-4}	2.54×10^{-2}
MAK	101795585	-1.54	8.68×10^{-4}	2.55×10^{-2}
LOC101800549	101800549	1.42	8.70×10^{-4}	2.55×10^{-2}
SWI5	101792721	-1.41	8.72×10^{-4}	2.55×10^{-2}
IP6K3	101790367	1.38	8.83×10^{-4}	2.57×10^{-2}
LOC101791983	101791983	1.67	9.02×10^{-4}	2.61×10^{-2}
TMEM117	101791823	1.41	9.03×10^{-4}	2.61×10^{-2}
KLHL38	101799560	1.38	9.05×10^{-4}	2.61×10^{-2}
GLB1L	101799366	-1.60	9.07×10^{-4}	2.61×10^{-2}
LOC101794131	101794131	-1.40	9.08×10^{-4}	2.61×10^{-2}
TPM2	101795171	1.38	9.15×10^{-4}	2.62×10^{-2}
KLHDC3	101804210	1.38	9.40×10^{-4}	2.69×10^{-2}
CNGA3	113839601	2.14	9.42×10^{-4}	2.69×10^{-2}
MEGF6	101791897	-1.45	9.44×10^{-4}	2.69×10^{-2}
SPATA18	101789394	-1.80	9.63×10^{-4}	2.73×10^{-2}
SCNN1G	101795334	1.41	9.98×10^{-4}	2.83×10^{-2}

UBTD1	119717302	1.54	1.01×10^{-3}	2.87×10^{-2}
LOC110352094	110352094	2.25	1.03×10^{-3}	2.91×10^{-2}
CIB2	101801602	1.41	1.04×10^{-3}	2.93×10^{-2}
NPR3	101802126	-1.45	1.05×10^{-3}	2.93×10^{-2}
GABRG2	101789533	1.39	1.05×10^{-3}	2.93×10^{-2}
SRRM4	101799810	1.58	1.05×10^{-3}	2.94×10^{-2}
IDUA	101792515	1.47	1.08×10^{-3}	3.01×10^{-2}
MLF1	101801360	1.36	1.11×10^{-3}	3.09×10^{-2}
LOC119713793	119713793	1.37	1.11×10^{-3}	3.09×10^{-2}
MPP7	101792425	1.37	1.12×10^{-3}	3.10×10^{-2}
CCN5	110352128	1.54	1.12×10^{-3}	3.10×10^{-2}
RASGRP3	101790483	1.37	1.13×10^{-3}	3.12×10^{-2}
SH2B2	101794843	-1.37	1.14×10^{-3}	3.12×10^{-2}
ITFG2	101800353	-1.35	1.19×10^{-3}	3.25×10^{-2}
SLC25A19	101803097	1.35	1.22×10^{-3}	3.33×10^{-2}
LOC119714765	119714765	-1.58	1.22×10^{-3}	3.33×10^{-2}
LOC113841751	113841751	-1.41	1.23×10^{-3}	3.34×10^{-2}
DOK5	101796412	1.36	1.26×10^{-3}	3.41×10^{-2}
LOC101801660	101801660	-1.76	1.28×10^{-3}	3.46×10^{-2}
PHTF2	101790512	1.34	1.29×10^{-3}	3.49×10^{-2}
LOC110353953	110353953	-1.34	1.30×10^{-3}	3.49×10^{-2}
TTC19	101799684	-1.34	1.34×10^{-3}	3.59×10^{-2}
LOC101804257	101804257	1.37	1.34×10^{-3}	3.59×10^{-2}
P2RY6	101799392	-1.78	1.35×10^{-3}	3.61×10^{-2}
SOCS2	101790013	1.50	1.36×10^{-3}	3.64×10^{-2}
ABHD5	119715932	1.33	1.38×10^{-3}	3.65×10^{-2}
NEURL1B	101799942	-1.41	1.38×10^{-3}	3.65×10^{-2}
ANKRD33B	101794598	-1.64	1.39×10^{-3}	3.69×10^{-2}
HTRA3	101790796	-1.39	1.40×10^{-3}	3.70×10^{-2}
TTBK1	101796937	1.45	1.42×10^{-3}	3.74×10^{-2}
DPH5	101793155	1.36	1.44×10^{-3}	3.78×10^{-2}
UBC	101797590	-1.32	1.48×10^{-3}	3.87×10^{-2}
UCK2	101798403	1.36	1.50×10^{-3}	3.93×10^{-2}
SFRP5	119717332	1.86	1.51×10^{-3}	3.94×10^{-2}
LOC119718341	119718341	-1.32	1.53×10^{-3}	3.97×10^{-2}
ULK1	101797231	1.31	1.54×10^{-3}	3.99×10^{-2}
TALDO1	101796720	1.34	1.54×10^{-3}	3.99×10^{-2}
SMIM32	106016818	-1.39	1.54×10^{-3}	3.99×10^{-2}
TECRL	101792252	-2.02	1.55×10^{-3}	4.01×10^{-2}
CFAP91	101802436	1.44	1.57×10^{-3}	4.03×10^{-2}
MYBPC3	101804232	-1.42	1.57×10^{-3}	4.04×10^{-2}
LOC101797680	101797680	-1.36	1.57×10^{-3}	4.04×10^{-2}
ADAM23	101799669	1.42	1.61×10^{-3}	4.12×10^{-2}
LOC119714653	119714653	1.76	1.61×10^{-3}	4.12×10^{-2}
LOC101790677	101790677	1.48	1.63×10^{-3}	4.15×10^{-2}

LOC110354432	110354432	1.68	1.66×10^{-3}	4.22×10^{-2}
LOC106019000	106019000	-1.60	1.67×10^{-3}	4.22×10^{-2}
VCAN	101795323	1.56	1.67×10^{-3}	4.22×10^{-2}
CAMKV	106018405	1.33	1.73×10^{-3}	4.37×10^{-2}
ABCA7	113845670	1.44	1.76×10^{-3}	4.42×10^{-2}
LOC119717912	119717912	-1.32	1.78×10^{-3}	4.47×10^{-2}
IGFBP5	101803411	1.34	1.81×10^{-3}	4.53×10^{-2}
LOC113840312	113840312	1.51	1.81×10^{-3}	4.53×10^{-2}
ASB4	101795945	1.30	1.82×10^{-3}	4.53×10^{-2}
IGF2BP2	101799699	-1.42	1.82×10^{-3}	4.53×10^{-2}
LOC101803957	101803957	1.36	1.84×10^{-3}	4.57×10^{-2}
LOC113839856	113839856	1.38	1.87×10^{-3}	4.64×10^{-2}
RPL3	101793495	1.29	1.89×10^{-3}	4.67×10^{-2}
ZDHHC16	119717296	1.30	1.90×10^{-3}	4.70×10^{-2}
LOC119716123	119716123	1.51	1.91×10^{-3}	4.70×10^{-2}
LOC101802407	101802407	-1.37	1.92×10^{-3}	4.72×10^{-2}
UBA7	101790099	-1.31	1.92×10^{-3}	4.72×10^{-2}
JDP2	101793146	1.33	1.94×10^{-3}	4.75×10^{-2}
FOXO1	101795447	1.29	2.04×10^{-3}	4.97×10^{-2}
