# Supplementary material for: Integrated miRNAs, Transcriptome, and Metabolome Uncover Underlying Mechanisms for Breast Muscle Metabolic Regulation in Liancheng White and Cherry Valley Ducks
Source: Animals (Basel). 2026 Mar 16;16(6):934. doi: 10.3390/ani16060934 (PMC13023296; doi:10.3390/ani16060934)
Supplement: Supplementary file 1 [file animals-16-00934-s001.zip › Table S5. Statistics of miRNA-seq quality control.pdf]

Table S5. Statistics of miRNA-seq quality control.

Library	Total_reads	N% > 10%	Low quality	5_adapter _contamine	3_adapter_nul l or insert_null	Reads with ployA/T/G/C	Clean reads
BD1	13628160 (100.00%)	1 (0.00%)	0 (0.00%)	869 (0.01%)	177254 (1.30%)	25335 (0.19%)	13424701 (98.51%)
BD2	11875752 (100.00%)	0 (0.00%)	0 (0.00%)	1186 (0.01%)	217427 (1.83%)	31021 (0.26%)	11626118 (97.90%)
BD3	11670727 (100.00%)	0 (0.00%)	0 (0.00%)	489 (0.00%)	204225 (1.75%)	17163 (0.15%)	11448850 (98.10%)
LD1	11380453 (100.00%)	0 (0.00%)	0 (0.00%)	1504 (0.01%)	165232 (1.45%)	26826 (0.24%)	11186891 (98.30%)
LD2	11713650 (100.00%)	0 (0.00%)	0 (0.00%)	761 (0.01%)	217412 (1.86%)	27240 (0.23%)	11468237 (97.90%)
LD3	11820680 (100.00%)	0 (0.00%)	0 (0.00%)	1333 (0.01%)	170657 (1.44%)	19368 (0.16%)	11629322 (98.38%)
