# Supplementary material for: Integrated miRNAs, Transcriptome, and Metabolome Uncover Underlying Mechanisms for Breast Muscle Metabolic Regulation in Liancheng White and Cherry Valley Ducks
Source: Animals (Basel). 2026 Mar 16;16(6):934. doi: 10.3390/ani16060934 (PMC13023296; doi:10.3390/ani16060934)
Supplement: Supplementary file 1 [file animals-16-00934-s001.zip › Table S6. Correlation analysis of miRNA-mRNA.pdf]

Table S6. Correlation analysis of miRNA-mRNA (cor < -0.3).

miRNA	Target_gene_ID	Target_gene_name	cor	*P* value
oan-miR-1386	101802351	ADAMTSL2	-0.32	0.54
oan-miR-1386	110354783	LOC110354783	-0.32	0.54
oan-miR-1386	101805281	MST1R	-0.33	0.52
ggo-miR-148a	101798492	LOC101798492	-0.34	0.51
hsa-miR-148a-3p	101798492	LOC101798492	-0.34	0.51
oan-miR-1386	101804756	LOC101804756	-0.35	0.50
mdo-miR-122-5p	101793146	JDP2	-0.35	0.49
oan-miR-1386	119718657	LOC119718657	-0.37	0.47
dre-miR-148	101798492	LOC101798492	-0.38	0.46
ola-miR-148	101798492	LOC101798492	-0.38	0.46
gga-miR-132b-5p	101798600	SLC4A4	-0.41	0.42
oan-miR-1386	101794631	LOC101794631	-0.41	0.41
oan-miR-1386	113843797	LOC113843797	-0.42	0.40
oan-miR-1386	101804210	KLHDC3	-0.46	0.36
novel_163	119716536	LOC119716536	-0.46	0.35
oan-miR-1386	101799143	NGEF	-0.46	0.35
oan-miR-1386	113840312	LOC113840312	-0.47	0.35
xtr-miR-184	101793601	BTG2	-0.49	0.32
oan-miR-1386	119717136	LOC119717136	-0.50	0.31
mdo-miR-122-5p	101790483	RASGRP3	-0.51	0.30
oan-miR-1386	101791200	SOCS3	-0.52	0.29
oan-miR-1386	101789781	RABEPK	-0.54	0.27
oan-miR-1386	119717606	SLC6A9	-0.55	0.26
oan-miR-1386	101803957	LOC101803957	-0.58	0.23
oan-miR-1386	101793146	JDP2	-0.61	0.20
oan-miR-1386	101801185	RASD1	-0.62	0.19
oan-miR-1386	119715617	LOC119715617	-0.64	0.17
oan-miR-1386	110352128	CCN5	-0.65	0.17
oan-miR-1386	113842967	LOC113842967	-0.66	0.15
novel_163	119713793	LOC119713793	-0.68	0.14
oan-miR-1386	101805082	LOC101805082	-0.72	0.11
novel_163	119717600	LOC119717600	-0.80	0.06
gga-miR-132b-5p	119713606	H4	-0.90	0.01
ocu-miR-16a-5p	101790717	DNAH9	-0.94	0.01
cja-miR-3065	101797091	LOC101797091	-0.83	0.04
hsa-miR-3065-3p	101797091	LOC101797091	-0.83	0.04
mmu-miR-3065-3p	101797091	LOC101797091	-0.83	0.04
cja-miR-3065	119714170	COPZ2	-0.82	0.05
hsa-miR-3065-3p	119714170	COPZ2	-0.82	0.05
mmu-miR-3065-3p	119714170	COPZ2	-0.82	0.05
mmu-miR-3065-3p	101802594	DMGDH	-0.79	0.06
oni-miR-17b	101794146	LOC101794146	-0.75	0.08

oan-miR-15c-5p	101792262	CCDC13	-0.69	0.13
pal-miR-454-3p	106019355	LOC106019355	-0.67	0.15
mmu-miR-3065-3p	119718222	RPUSD3	-0.64	0.17
mmu-miR-3065-3p	113843893	LOC113843893	-0.63	0.18
sha-miR-19b	101793162	CPXM2	-0.62	0.19
mmu-miR-199a-3p	101790078	P2RX6	-0.60	0.20
oni-miR-17b	110352460	LOC110352460	-0.59	0.22
mmu-miR-3065-3p	113845527	QRICH2	-0.59	0.22
gga-miR-106-5p	119715393	LOC119715393	-0.57	0.23
sme-miR-133a-3p	106019907	LOC106019907	-0.56	0.25
mmu-miR-3065-3p	101797680	LOC101797680	-0.55	0.26
oan-miR-15c-5p	101804291	LOC101804291	-0.52	0.29
cja-miR-3065	119713596	EXTL1	-0.51	0.30
hsa-miR-3065-3p	119713596	EXTL1	-0.51	0.30
mmu-miR-3065-3p	119713596	EXTL1	-0.51	0.30
cja-miR-3065	119713003	LOC119713003	-0.49	0.33
hsa-miR-3065-3p	119713003	LOC119713003	-0.49	0.33
mmu-miR-3065-3p	119713003	LOC119713003	-0.49	0.33
mmu-miR-3065-3p	101794339	LOC101794339	-0.48	0.33
sme-miR-133a-3p	119715393	LOC119715393	-0.47	0.34
oni-miR-17b	101797335	RASL10B	-0.44	0.39
oni-miR-17b	101804495	RPH3AL	-0.41	0.41
hsa-miR-3065-3p	113840577	LOC113840577	-0.40	0.43
sme-miR-133a-3p	119718222	RPUSD3	-0.39	0.45
mmu-miR-3065-3p	101799366	GLB1L	-0.38	0.45
sme-miR-133a-3p	101797147	LOC101797147	-0.38	0.46
