# Supplementary material for: Integrated miRNAs, Transcriptome, and Metabolome Uncover Underlying Mechanisms for Breast Muscle Metabolic Regulation in Liancheng White and Cherry Valley Ducks
Source: Animals (Basel). 2026 Mar 16;16(6):934. doi: 10.3390/ani16060934 (PMC13023296; doi:10.3390/ani16060934)
Supplement: Supplementary file 1 [file animals-16-00934-s001.zip › Table S7. Up target DEG_go_enrich (down DE miRNA).pdf]

Table S7. Up target DEG_go_enrich (down DE miRNA).

ONTOLOGY	ID	Description	Gene Ratio	Bg Ratio	Rich Factor	Fold Enrichment	zScore	*P* value	Gene name	Count
MF	GO:0140359	ABC-type transporter activity	3/16	43/12518	0.07	54.58	12.59	2.05×10^{-5}	LOC101797091/LOC101797680/LOC101794339	3
MF	GO:0042626	ATPase-coupled transmembrane transporter activity	3/16	82/12518	0.04	28.62	8.98	1.43×10^{-4}	LOC101797091/LOC101797680/LOC101794339	3
MF	GO:0015399	Primary active transmembrane transporter activity	3/16	88/12518	0.03	26.67	8.65	1.76×10^{-4}	LOC101797091/LOC101797680/LOC101794339	3
MF	GO:0042887	Amide transmembrane transporter activity	2/16	21/12518	0.10	74.51	12.06	3.17×10^{-4}	LOC101797091/LOC101797680	2
MF	GO:0140657	ATP-dependent activity	4/16	460/12518	0.01	6.80	4.54	2.30×10^{-3}	DNAH9/LOC101797091/LOC101797680/LOC101794339	4
MF	GO:0022804	Active transmembrane transporter activity	3/16	219/12518	0.01	10.72	5.19	2.50×10^{-3}	LOC101797091/LOC101797680/LOC101794339	3
MF	GO:0017111	Ribonucleoside triphosphate phosphatase activity	4/16	498/12518	0.01	6.28	4.30	3.07×10^{-3}	LOC101797091/LOC101797680/LOC101794339/RASL10B	4
MF	GO:0016887	ATP hydrolysis activity	3/16	237/12518	0.01	9.90	4.95	3.13×10^{-3}	LOC101797091/LOC101797680/LOC101794339	3
BP	GO:0014074	Response to purine-containing compound	1/14	10/11649	0.10	83.21	9.02	1.20×10^{-2}	P2RX6	1
CC	GO:0030126	COPI vesicle coat	1/13	11/11407	0.09	79.77	8.83	1.25×10^{-2}	COPZ2	1
CC	GO:0030663	COPI-coated vesicle membrane	1/13	11/11407	0.09	79.77	8.83	1.25×10^{-2}	COPZ2	1
CC	GO:0034451	Centriolar satellite	1/13	11/11407	0.09	79.77	8.83	1.25×10^{-2}	CCDC13	1
BP	GO:0046683	response to organophosphorus	1/14	11/11649	0.09	75.64	8.59	1.31×10^{-2}	P2RX6	1
CC	GO:0030137	COPI-coated vesicle	1/13	12/11407	0.08	73.12	8.44	1.36×10^{-2}	COPZ2	1
MF	GO:0008569	Minus-end-directed microtubule motor activity	1/16	13/12518	0.08	60.18	7.64	1.65×10^{-2}	DNAH9	1
MF	GO:0009982	Pseudouridine synthase activity	1/16	13/12518	0.08	60.18	7.64	1.65×10^{-2}	RPUSD3	1
BP	GO:0001522	Pseudouridine synthesis	1/14	15/11649	0.07	55.47	7.32	1.79×10^{-2}	RPUSD3	1
BP	GO:0048002	Antigen processing and presentation of	1/14	18/11649	0.06	46.23	6.66	2.14×10^{-2}	LOC101797091	1

		peptide antigen								
CC	GO:0005637	Nuclear inner membrane	1/13	20/11407	0.05	43.87	6.48	2.26×10^{-2}	P2RX6	1
MF	GO:0001614	Purinergic nucleotide receptor activity	1/16	20/12518	0.05	39.12	6.10	2.53×10^{-2}	P2RX6	1
MF	GO:0016502	Nucleotide receptor activity	1/16	20/12518	0.05	39.12	6.10	2.53×10^{-2}	P2RX6	1
BP	GO:1905515	Non-motile cilium assembly	1/14	23/11649	0.04	36.18	5.86	2.73×10^{-2}	CCDC13	1
CC	GO:0030660	Golgi-associated vesicle membrane	1/13	25/11407	0.04	35.10	5.76	2.81×10^{-2}	COPZ2	1
CC	GO:0031965	Nuclear membrane	1/13	25/11407	0.04	35.10	5.76	2.81×10^{-2}	P2RX6	1
BP	GO:0006891	Intra-Golgi vesicle-mediated transport	1/14	24/11649	0.04	34.67	5.73	2.85×10^{-2}	COPZ2	1
MF	GO:0004181	Metallocarboxypeptidase activity	1/16	23/12518	0.04	34.02	5.67	2.90×10^{-2}	CPXM2	1
MF	GO:0051959	Dynein light intermediate chain binding	1/16	23/12518	0.04	34.02	5.67	2.90×10^{-2}	DNAH9	1
BP	GO:0006890	retrograde Vesicle-mediated transport, Golgi to endoplasmic reticulum	1/14	25/11649	0.04	33.28	5.60	2.96×10^{-2}	COPZ2	1
BP	GO:0019882	Antigen processing and presentation	1/14	25/11649	0.04	33.28	5.60	2.96×10^{-2}	LOC101797091	1
BP	GO:0031122	Cytoplasmic microtubule organization	1/14	25/11649	0.04	33.28	5.60	2.96×10^{-2}	CCDC13	1
MF	GO:0016866	Intramolecular transferase activity	1/16	24/12518	0.04	32.60	5.54	3.03×10^{-2}	RPUSD3	1
MF	GO:0003725	Double-stranded RNA binding	1/16	25/12518	0.04	31.30	5.42	3.15×10^{-2}	LOC101804291	1
BP	GO:0006334	Nucleosome assembly	1/14	27/11649	0.04	30.82	5.38	3.20×10^{-2}	LOC101797147	1
MF	GO:0005231	Excitatory extracellular ligand-gated monoatomic ion channel activity	1/16	26/12518	0.04	30.09	5.31	3.27×10^{-2}	P2RX6	1
MF	GO:0004180	Carboxypeptidase activity	1/16	29/12518	0.03	26.98	5.01	3.65×10^{-2}	CPXM2	1
MF	GO:0045505	Dynein intermediate chain binding	1/16	30/12518	0.03	26.08	4.92	3.77×10^{-2}	DNAH9	1
BP	GO:0034728	Nucleosome organization	1/14	33/11649	0.03	25.21	4.83	3.90×10^{-2}	LOC101797147	1
CC	GO:0005798	Golgi-associated vesicle	1/13	36/11407	0.03	24.37	4.74	4.03×10^{-2}	COPZ2	1
MF	GO:0000049	tRNA binding	1/16	33/12518	0.03	23.71	4.67	4.14×10^{-2}	LOC101804291	1
CC	GO:0030286	Dynein complex	1/13	41/11407	0.02	21.40	4.42	4.58×10^{-2}	DNAH9	1

BP	GO:0006886	Intracellular protein transport	2/14	289/11649	0.01	5.76	2.84	4.59×10^{-2}	COPZ2/RPH3AL	2
CC	GO:0030120	Vesicle coat	1/13	43/11407	0.02	20.41	4.31	4.79×10^{-2}	COPZ2	1
