# Supplementary material for: Integrated miRNAs, Transcriptome, and Metabolome Uncover Underlying Mechanisms for Breast Muscle Metabolic Regulation in Liancheng White and Cherry Valley Ducks
Source: Animals (Basel). 2026 Mar 16;16(6):934. doi: 10.3390/ani16060934 (PMC13023296; doi:10.3390/ani16060934)
Supplement: Supplementary file 1 [file animals-16-00934-s001.zip › Table S8. Down target DEG_go_enrich (up DE miRNA).pdf]

Table S8. Down target DEG_go_enrich (up DE miRNA).

ONTOLOGY	ID	Description	Gene Ratio	Bg Ratio	Rich Factor	Fold Enrichment	zScore	*P* value	Gene name	Count
MF	GO:0015370	Solute:sodium symporter activity	2/20	48/12518	0.04	26.08	6.96	2.62×10^{-3}	SLC4A4/SLC6A9	2
BP	GO:0043085	Positive regulation of catalytic activity	3/16	219/11649	0.01	9.97	4.97	3.06×10^{-3}	MST1R/NGEF/RASGRP3	3
MF	GO:0015294	Solute:monoatomic cation symporter activity	2/20	59/12518	0.03	21.22	6.23	3.93×10^{-3}	SLC4A4/SLC6A9	2
MF	GO:0015293	Symporter activity	2/20	65/12518	0.03	19.26	5.90	4.75×10^{-3}	SLC4A4/SLC6A9	2
MF	GO:0022804	Active transmembrane transporter activity	3/20	219/12518	0.01	8.57	4.52	4.83×10^{-3}	LOC119718657/SLC4A4/SLC6A9	3
BP	GO:0044093	Positive regulation of molecular function	3/16	260/11649	0.01	8.40	4.48	4.96×10^{-3}	MST1R/NGEF/RASGRP3	3
BP	GO:0043547	Positive regulation of GTPase activity	2/16	101/11649	0.02	14.42	5.02	8.25×10^{-3}	NGEF/RASGRP3	2
MF	GO:0015081	Sodium ion transmembrane transporter activity	2/20	97/12518	0.02	12.91	4.71	1.03×10^{-2}	SLC4A4/SLC6A9	2
BP	GO:0051345	Positive regulation of hydrolase activity	2/16	118/11649	0.02	12.34	4.59	1.11×10^{-2}	NGEF/RASGRP3	2
BP	GO:0015711	Organic anion transport	2/16	124/11649	0.02	11.74	4.46	1.22×10^{-2}	SLC4A4/SLC6A9	2
BP	GO:0050790	Regulation of catalytic activity	3/16	377/11649	0.01	5.79	3.51	1.38×10^{-2}	MST1R/NGEF/RASGRP3	3
BP	GO:0043087	Regulation of GTPase activity	2/16	135/11649	0.01	10.79	4.24	1.44×10^{-2}	NGEF/RASGRP3	2
MF	GO:0022853	Active monoatomic ion transmembrane transporter activity	2/20	124/12518	0.02	10.10	4.07	1.65×10^{-2}	SLC4A4/SLC6A9	2
MF	GO:0015291	Secondary active transmembrane transporter activity	2/20	130/12518	0.02	9.63	3.96	1.80×10^{-2}	SLC4A4/SLC6A9	2
MF	GO:0005452	Solute:inorganic anion antiporter activity	1/20	12/12518	0.08	52.16	7.09	1.90×10^{-2}	SLC4A4	1
MF	GO:0005085	Guanyl-nucleotide exchange factor activity	2/20	139/12518	0.01	9.01	3.80	2.04×10^{-2}	NGEF/RASGRP3	2
BP	GO:1902475	L-alpha-amino acid transmembrane transport	1/16	15/11649	0.07	48.54	6.83	2.04×10^{-2}	SLC6A9	1
MF	GO:0005283	Amino acid:sodium symporter activity	1/20	13/12518	0.08	48.15	6.80	2.06×10^{-2}	SLC6A9	1
MF	GO:0008514	Organic anion transmembrane transporter activity	2/20	143/12518	0.01	8.75	3.73	2.15×10^{-2}	SLC4A4/SLC6A9	2
MF	GO:0005416	Amino acid:monoatomic cation symporter activity	1/20	14/12518	0.07	44.71	6.55	2.21×10^{-2}	SLC6A9	1
MF	GO:00	1-phosphatidylinosit	1/20	14/12518	0.07	44.71	6.55	2.21×10^{-2}	SOCS3	1

	46935	ol-3-kinase regulator activity								
MF	GO:0015106	Bicarbonate transmembrane transporter activity	1/20	15/12518	0.07	41.73	6.31	2.37×10^{-2}	SLC4A4	1
BP	GO:0015804	Neutral amino acid transport	1/16	19/11649	0.05	38.32	6.04	2.58×10^{-2}	SLC6A9	1
BP	GO:0065009	Regulation of molecular function	3/16	479/11649	0.01	4.56	2.95	2.60×10^{-2}	MST1R/NGEF/RASGRP3	3
BP	GO:0015807	L-amino acid transport	1/16	20/11649	0.05	36.40	5.88	2.71×10^{-2}	SLC6A9	1
BP	GO:0030517	Negative regulation of axon extension	1/16	20/11649	0.05	36.40	5.88	2.71×10^{-2}	LOC101805082	1
BP	GO:0048841	Regulation of axon extension involved in axon guidance	1/16	20/11649	0.05	36.40	5.88	2.71×10^{-2}	LOC101805082	1
BP	GO:0048843	Negative regulation of axon extension involved in axon guidance	1/16	20/11649	0.05	36.40	5.88	2.71×10^{-2}	LOC101805082	1
BP	GO:0048846	Axon extension involved in axon guidance	1/16	20/11649	0.05	36.40	5.88	2.71×10^{-2}	LOC101805082	1
BP	GO:0050771	Negative regulation of axonogenesis	1/16	20/11649	0.05	36.40	5.88	2.71×10^{-2}	LOC101805082	1
BP	GO:0050922	Negative regulation of chemotaxis	1/16	20/11649	0.05	36.40	5.88	2.71×10^{-2}	LOC101805082	1
BP	GO:0051453	Regulation of intracellular pH	1/16	20/11649	0.05	36.40	5.88	2.71×10^{-2}	SLC4A4	1
BP	GO:1902284	Neuron projection extension involved in neuron projection guidance	1/16	20/11649	0.05	36.40	5.88	2.71×10^{-2}	LOC101805082	1
CC	GO:0031012	Extracellular matrix	2/18	168/11407	0.01	7.54	3.40	2.83×10^{-2}	ADAMTSL2/CCN5	2
BP	GO:0050768	Negative regulation of neurogenesis	1/16	21/11649	0.05	34.67	5.73	2.85×10^{-2}	LOC101805082	1
BP	GO:0050919	Negative chemotaxis	1/16	21/11649	0.05	34.67	5.73	2.85×10^{-2}	LOC101805082	1
BP	GO:0051961	Negative regulation of nervous system development	1/16	21/11649	0.05	34.67	5.73	2.85×10^{-2}	LOC101805082	1
CC	GO:0030312	External encapsulating structure	2/18	169/11407	0.01	7.50	3.38	2.86×10^{-2}	ADAMTSL2/CCN5	2
BP	GO:0010977	Negative regulation of neuron projection development	1/16	22/11649	0.05	33.09	5.59	2.98×10^{-2}	LOC101805082	1
BP	GO:0030308	Negative regulation of cell growth	1/16	22/11649	0.05	33.09	5.59	2.98×10^{-2}	LOC101805082	1
BP	GO:0030641	Regulation of cellular pH	1/16	22/11649	0.05	33.09	5.59	2.98×10^{-2}	SLC4A4	1
CC	GO:1902710	GABA receptor complex	1/18	20/11407	0.05	31.69	5.46	3.11×10^{-2}	LOC101798492	1
CC	GO:1902711	GABA-A receptor complex	1/18	20/11407	0.05	31.69	5.46	3.11×10^{-2}	LOC101798492	1
BP	GO:0048640	Negative regulation of developmental	1/16	23/11649	0.04	31.65	5.46	3.11×10^{-2}	LOC101805082	1

		growth								
MF	GO:0004890	GABA-A receptor activity	1/20	20/12518	0.05	31.30	5.42	3.15×10^{-2}	LOC101798492	1
MF	GO:0005343	Organic acid:sodium symporter activity	1/20	20/12518	0.05	31.30	5.42	3.15×10^{-2}	SLC6A9	1
MF	GO:0030215	Semaphorin receptor binding	1/20	20/12518	0.05	31.30	5.42	3.15×10^{-2}	LOC101805082	1
CC	GO:0043235	Receptor complex	1/18	179/11407	0.01	7.08	3.26	3.18×10^{-2}	MST1R/LOC101798492	2
BP	GO:0051336	Regulation of hydrolase activity	2/16	209/11649	0.01	6.97	3.23	3.26×10^{-2}	NGEF/RASGRP3	2
MF	GO:0042887	Amide transmembrane transporter activity	1/20	21/12518	0.05	29.80	5.28	3.30×10^{-2}	LOC119718657	1
BP	GO:0098657	Import into cell	2/16	211/11649	0.01	6.90	3.21	3.32×10^{-2}	MST1R/SLC6A9	2
BP	GO:0001755	Neural crest cell migration	1/16	25/11649	0.04	29.12	5.22	3.38×10^{-2}	LOC101805082	1
BP	GO:0030516	Regulation of axon extension	1/16	25/11649	0.04	29.12	5.22	3.38×10^{-2}	LOC101805082	1
BP	GO:0031345	Negative regulation of cell projection organization	1/16	25/11649	0.04	29.12	5.22	3.38×10^{-2}	LOC101805082	1
BP	GO:0045926	Negative regulation of growth	1/16	25/11649	0.04	29.12	5.22	3.38×10^{-2}	LOC101805082	1
BP	GO:0090497	Mesenchymal cell migration	1/16	25/11649	0.04	29.12	5.22	3.38×10^{-2}	LOC101805082	1
BP	GO:0007268	Chemical synaptic transmission	2/16	220/11649	0.01	6.62	3.12	3.58×10^{-2}	LOC101798492/SLC6A9	2
BP	GO:0098916	Anterograde trans-synaptic signaling	2/16	220/11649	0.01	6.62	3.12	3.58×10^{-2}	LOC101798492/SLC6A9	2
BP	GO:0099537	Trans-synaptic signaling	2/16	220/11649	0.01	6.62	3.12	3.58×10^{-2}	LOC101798492/SLC6A9	2
MF	GO:0016917	GABA receptor activity	1/20	23/12518	0.04	27.21	5.03	3.61×10^{-2}	LOC101798492	1
MF	GO:0045499	Chemorepellent activity	1/20	23/12518	0.04	27.21	5.03	3.61×10^{-2}	LOC101805082	1
BP	GO:0006334	Nucleosome assembly	1/16	27/11649	0.04	26.97	5.01	3.65×10^{-2}	H4	1
BP	GO:0010721	Negative regulation of cell development	1/16	27/11649	0.04	26.97	5.01	3.65×10^{-2}	LOC101805082	1
BP	GO:0014032	Neural crest cell development	1/16	27/11649	0.04	26.97	5.01	3.65×10^{-2}	LOC101805082	1
BP	GO:0014033	Neural crest cell differentiation	1/16	27/11649	0.04	26.97	5.01	3.65×10^{-2}	LOC101805082	1
BP	GO:0048863	Stem cell differentiation	1/16	27/11649	0.04	26.97	5.01	3.65×10^{-2}	LOC101805082	1
BP	GO:0048864	Stem cell development	1/16	27/11649	0.04	26.97	5.01	3.65×10^{-2}	LOC101805082	1
BP	GO:0061387	Regulation of extent of cell growth	1/16	27/11649	0.04	26.97	5.01	3.65×10^{-2}	LOC101805082	1
BP	GO:0071526	Semaphorin-plexin signaling pathway	1/16	27/11649	0.04	26.97	5.01	3.65×10^{-2}	LOC101805082	1
CC	GO:0005942	Phosphatidylinositol 3-kinase complex	1/18	25/11407	0.04	25.35	4.85	3.88×10^{-2}	SOCS3	1
BP	GO:0008285	Negative regulation of cell population	1/16	29/11649	0.03	25.11	4.82	3.91×10^{-2}	BTG2	1

		proliferation								
BP	GO:0048762	Mesenchymal cell differentiation	1/16	29/11649	0.03	25.11	4.82	3.91×10^{-2}	LOC101805082	1
BP	GO:0060485	Mesenchyme development	1/16	29/11649	0.03	25.11	4.82	3.91×10^{-2}	LOC101805082	1
BP	GO:0099536	Synaptic signaling	2/16	231/11649	0.01	6.30	3.02	3.91×10^{-2}	LOC101798492/SLC6A9	2
MF	GO:0008201	Heparin binding	1/20	25/12518	0.04	25.04	4.81	3.92×10^{-2}	CCN5	1
MF	GO:0015175	Neutral L-amino acid transmembrane transporter activity	1/20	25/12518	0.04	25.04	4.81	3.92×10^{-2}	SLC6A9	1
CC	GO:0016323	Basolateral plasma membrane	1/18	26/11407	0.04	24.37	4.74	4.03×10^{-2}	SLC4A4	1
BP	GO:0015695	Organic cation transport	1/16	30/11649	0.03	24.27	4.73	4.04×10^{-2}	SLC6A9	1
BP	GO:0048675	Axon extension	1/16	30/11649	0.03	24.27	4.73	4.04×10^{-2}	LOC101805082	1
BP	GO:1905039	Carboxylic acid transmembrane transport	1/16	30/11649	0.03	24.27	4.73	4.04×10^{-2}	SLC6A9	1
BP	GO:1903825	Organic acid transmembrane transport	1/16	31/11649	0.03	23.49	4.65	4.18×10^{-2}	SLC6A9	1
BP	GO:0006885	Regulation of pH	1/16	32/11649	0.03	22.75	4.57	4.31×10^{-2}	SLC4A4	1
BP	GO:0050920	Regulation of chemotaxis	1/16	32/11649	0.03	22.75	4.57	4.31×10^{-2}	LOC101805082	1
BP	GO:0090630	Activation of GTPase activity	1/16	32/11649	0.03	22.75	4.57	4.31×10^{-2}	NGEF	1
BP	GO:0034728	Nucleosome organization	1/16	33/11649	0.03	22.06	4.49	4.44×10^{-2}	H4	1
BP	GO:0040013	Negative regulation of locomotion	1/16	33/11649	0.03	22.06	4.49	4.44×10^{-2}	LOC101805082	1
BP	GO:0016477	Cell migration	2/16	250/11649	0.01	5.82	2.86	4.52×10^{-2}	MST1R/LOC101805082	2
BP	GO:0001667	Ameboidal-type cell migration	1/16	34/11649	0.03	21.41	4.42	4.57×10^{-2}	LOC101805082	1
BP	GO:0045596	Negative regulation of cell differentiation	1/16	34/11649	0.03	21.41	4.42	4.57×10^{-2}	LOC101805082	1
CC	GO:0009925	Basal plasma membrane	1/18	30/11407	0.03	21.12	4.39	4.63×10^{-2}	SLC4A4	1
BP	GO:0001558	Regulation of cell growth	1/16	35/11649	0.03	20.80	4.35	4.70×10^{-2}	LOC101805082	1
BP	GO:0048638	Regulation of developmental growth	1/16	35/11649	0.03	20.80	4.35	4.70×10^{-2}	LOC101805082	1
BP	GO:0006909	Phagocytosis	1/16	36/11649	0.03	20.22	4.28	4.83×10^{-2}	MST1R	1
BP	GO:0046854	Phosphatidylinositol phosphate biosynthetic process	1/16	36/11649	0.03	20.22	4.28	4.83×10^{-2}	SOCS3	1
BP	GO:1990138	Neuron projection extension	1/16	36/11649	0.03	20.22	4.28	4.83×10^{-2}	LOC101805082	1
CC	GO:0045178	Basal part of cell	1/18	32/11407	0.03	19.80	4.23	4.93×10^{-2}	SLC4A4	1
BP	GO:0008361	Regulation of cell size	1/16	37/11649	0.03	19.68	4.22	4.97×10^{-2}	LOC101805082	1
