# Supplementary material for: Integrated miRNAs, Transcriptome, and Metabolome Uncover Underlying Mechanisms for Breast Muscle Metabolic Regulation in Liancheng White and Cherry Valley Ducks
Source: Animals (Basel). 2026 Mar 16;16(6):934. doi: 10.3390/ani16060934 (PMC13023296; doi:10.3390/ani16060934)
Supplement: Supplementary file 1 [file animals-16-00934-s001.zip › Table S9. Correlation analysis of DAMs and DEGs.pdf]

Table S9. Correlation analysis of DAMs and DEGs.

DAMs	DEGs	r	*P* value
2'-Deoxyuridine	SOCS3	-0.953894067	0.003139631
Alpha-D-Glucose	SOCS3	-0.923940941	0.008457471
2'-Deoxyuridine	LOC119718657	-0.911152692	0.011490093
L-Methionine	DMGDH	-0.90587351	0.012872723
L-Threonine	DNAH9	-0.870667987	0.024008501
Taurochenodeoxycholate	DMGDH	-0.870188318	0.024182876
Taurine	DNAH9	-0.864382436	0.026341038
L-Methionine	DNAH9	-0.854616039	0.030168291
Alpha-D-Glucose	LOC119718657	-0.85412157	0.03036859
L-Threonine	LOC101797680	-0.85057111	0.031825191
L-Carnitine	SOCS3	-0.832335479	0.039810446
Alpha-D-Glucose	H4	-0.826309311	0.042632693
Alpha-D-Glucose	LOC101797091	0.814720935	0.048312337
Alpha-D-Glucose	DMGDH	0.816285962	0.047526019
Taurochenodeoxycholate	H4	0.827339585	0.042143785
L-Valine	SOCS3	0.828078449	0.041794785
Glycine	SLC4A4	0.831957337	0.039984882
L-Leucine	SOCS3	0.838555505	0.036992517
L-Threonine	LOC101798492	0.844828344	0.034249234
L-Threonine	LOC119718657	0.845750339	0.033854409
L-Arginine	SLC6A9	0.855120842	0.029964451
Taurochenodeoxycholate	LOC119718657	0.856773323	0.029301754
Glycine	SLC6A9	0.857452554	0.029031396
2'-Deoxyuridine	DMGDH	0.857488218	0.029017233
L-Arginine	SLC4A4	0.868806156	0.024688694
Taurine	SLC4A4	0.87438182	0.022678768
2'-Deoxyuridine	DNAH9	0.881983323	0.02007004
L-Methionine	SLC6A9	0.888423095	0.017979575
L-Methionine	LOC119718657	0.900016568	0.014495278
L-Threonine	SLC4A4	0.922934745	0.008679733
L-Methionine	SOCS3	0.923849026	0.008477658
Inosine	SLC6A9	0.938378309	0.005578853
L-Glutamine	SLC4A4	0.945291467	0.004407663
Taurochenodeoxycholate	SOCS3	0.949208278	0.003804182
L-Threonine	SLC6A9	0.95048306	0.003617185
Inosine	SLC4A4	0.953397478	0.003207087
DL-Serine	SLC4A4	0.965585927	0.001756114
Taurine	SLC6A9	0.982253993	0.000469587
